# Hippo Pathway Regulation and Microenvironmental Cues Governing Human MSC Transition to Cancer‐Associated Fibroblasts

**DOI:** 10.1002/jcp.70219

**Published:** 2026-08-05

**Authors:** Pimjai Chingsuwanrote, Chanchao Lorthongpanich, Pakpoom Kheolamai, Ting Gang Chew, Chuti Laowtammathron, Sudjit Luanpitpong, Rangsun Parnpai, Surapol Issaragrisil

**Affiliations:** ^1^ Department of Medicine, Siriraj Center of Excellence for Stem Cell Research, Faculty of Medicine Siriraj Hospital Mahidol University Bangkok Thailand; ^2^ Mechanobiology and Stem Cell Fate Research Group, Faculty of Medicine Siriraj Hospital Mahidol University Bangkok Thailand; ^3^ Center of Excellence in Stem Cell Research and Innovation, Faculty of Medicine Thammasat University Pathumthani Thailand; ^4^ Department of Cardiology of the Second Affiliated Hospital, Zhejiang University School of Medicine Zhejiang University Hangzhou China; ^5^ The Zhejiang University‐University of Edinburgh Institute, Zhejiang University School of Medicine Zhejiang University Haining China; ^6^ School of Biotechnology, Institute of Agricultural Technology, Embryo Technology and Stem Cell Research Center Suranaree University of Technology Nakhon Ratchasima Thailand; ^7^ Bangkok Hematology Center, Wattanosoth Hospital BDMS Center of Excellence for Cancer Bangkok Thailand

**Keywords:** 3‐dimensional (3D) culture, cancer associated fibroblasts, hypoxic condition, mesenchymal stem cells, YAP

## Abstract

Cancer‐associated fibroblasts (CAFs) are key drivers of tumor progression. This study examined how three‐dimensional (3D) culture, hypoxia, and cancer‐derived soluble factors influence the transformation of human mesenchymal stem cells (hMSCs) into inflammatory CAFs (iCAFs). hMSCs from bone marrow, placenta, and chorion were cultured in 2D, in Matrigel‐based 3D systems, under hypoxia, and with soluble factors from colon cancer cells (HT29, HCT116). 3D culture strongly induced iCAF markers (IL1α, CSF3, IL6) while reducing myofibroblastic CAF markers (CCN2, MYL9, TAGLN). Hypoxia and cancer factors further enhanced this phenotype, promoting IL1α/IL6 secretion and shifting their influence from suppressing to stimulating cancer cell growth and angiogenesis. Mechanistically, these changes were associated with YAP/TAZ down‐regulation, and genetic depletion of YAP/TAZ alone was sufficient to convert hMSCs into iCAFs even in 2D culture. These findings highlight YAP/TAZ as critical regulators of hMSC‐to‐CAF transformation, with implications for therapeutic strategies targeting tumor stroma.

**Abbreviations**
3D: Three‐dimensional
CAF: Cancer Associated Fibroblast
CM: Conditioned medium
ECM: Extracellular Matrix
hMSCs: Human Mesenchymal Stem Cells
YAP: Yes‐Associated Protein

## 1 Introduction

Cancer‐associated fibroblasts (CAFs) have emerged as a critical component of the tumor microenvironment, which play an important role in regulating tumor progression and therapeutic outcomes (Fiori et al. 2019). CAFs facilitate tumor invasion and metastasis through the production of various components of the ECM and soluble factors that alter tissue architecture, suppress tumor immunosurveillance by creating an immunosuppressive environment, and secrete pro‐angiogenic factors that promote the formation of new blood vessels (Wu et al. 2021; Loh and Ma 2021).

Many previous studies show that one of the main sources of cancer‐associated fibroblasts (CAFs) is mesenchymal stem cells (Loh and Ma 2021; Borriello et al. 2017). Human mesenchymal stem cells (hMSCs) are multipotent stromal cells capable of differentiating into various cell types, including osteoblasts, chondrocytes, and adipocytes (Marion and Mao 2006; Lorthongpanich et al. 2024, 2021, 2019). These cells can be isolated from many tissues, such as bone marrow, adipose tissue, and the umbilical cord, and play a vital role in tissue repair and regeneration (Stefańska et al. 2020; Pittenger et al. 2019). In the context of cancer, hMSCs can transform into CAFs under the influence of the tumor microenvironment and contribute to tumor progression and resistance to treatment (Miyazaki et al. 2021; Antoon et al. 2024), by enhancing cancer cell survival, promoting cancer growth, and inhibiting tumor immunosurveillance. Despite their critical roles in cancer progression, the extracellular factors and intracellular signaling pathways that regulate the transformation of hMSCs into CAFs have not yet been fully understood.

Previous studies show that certain culture components, such as the extracellular matrix (ECM), and hypoxic condition could play a significant role in modulating MSC behavior and their transformation into pro‐tumorigenic CAFs (Cirri and Chiarugi 2012; Zhao et al. 2023). There is also evidence suggesting that the Hippo signaling pathway, whose dysregulation has been shown to affect the proliferation, differentiation, and functions of various cancers and cancer stromal cells, including CAFs, might also play an important role in this transformation process (Fang et al. 2023; Thinyakul et al. 2024).

Based on this information, this study, therefore, aimed to investigate the effect of various factors, including three‐dimensional culture in Matrigel, hypoxia, and cancer‐derived soluble factors on the transformation of bone marrow‐and placenta‐derived hMSCs (BM‐hMSCs and PL‐hMSCs) to CAFs. We also investigated the role of the Hippo signaling pathway in this transformation process by modulating the expression level of YAP and TAZ, the main effectors of this signaling, in hMSCs using genetic manipulation and determining the effects of these transformed hMSCs on cancer cell proliferation and migration compared to their wild‐type counterparts. This research not only improves our understanding of the transformation process of MSCs into CAFs but also explores the possibility of developing new cancer treatments by inhibiting this process and reducing the tumor‐supporting functions of these CAFs, thereby slowing tumor growth and improving the effectiveness of existing cancer treatments.

## 2 Materials and Methods

### 2.1 Cell Lines and Cell Culture

The immortalized BM‐hMSCs, UE6E7T‐3 (RRID: CVCL_3237), and immortalized human umbilical vein endothelial cells, HUEHT‐1 (RRID:CVCL_4W51), were purchased from JCRB (1136 (Mori et al. 2005) and 1458 (Anno et al. 2007)). All culture media and FBS were purchased from Gibco (ThermoFisher Scientific, Waltham, MA, USA). Primary CH‐hMSCs and PL‐hMSCs were derived from human chorionic and placenta tissues as previously described (Lorthongpanich et al. 2024). Two human colorectal cancer cell lines, HCT116 (RRID:CVCL_0291) and HT29 (RRID:CVCL_0320), were purchased from ATCC (CCL‐247 (Brattain et al. 1981) and HTB‐38 (Fogh and Trempe 1975)). All hMSCs cells were cultured in Dulbecco's modified eagle medium (DMEM) supplemented with 10% (v/v) fetal bovine serum (FBS), HCT116 cells were cultured in RPMI‐1640 supplemented with 10% (v/v) FBS, while HT29 were cultured in DMEM/F12 supplemented with 10% (v/v) FBS. For all normoxic cultures, cells were cultured in a humidified atmosphere containing 21% O_2_ and 5% CO_2_ at 37°C. For hypoxic cultures, cells were cultured in a humidified atmosphere containing 5% O_2_ and 5% CO_2_ at 37°C. The culture medium was replaced every 3 days. The authentication of UE6E7T‐3 (RRID: CVCL_3237) was confirmed by STR analysis.

### 2.2 RNA Isolation and Quantitative Real‐Time PCR

Total RNA was isolated using TRIzol (Invitrogen, Carlsbad, CA, USA). RNA were converted to cDNA using the RevertAid First Strand cDNA synthesis kit (Thermo Fisher, Waltham, MA, USA) according to the manufacturer's instructions, briefly, 300 ng of RNA was mixed with 100 pmol of oligo(dT)18 primer and incubated at 65°C for 5 min, followed by cooling on ice for 1 min. Subsequently, a master mix containing 1× reaction buffer, 1 U RNase inhibitor, 20 nmol dNTPs, and 200 U reverse transcriptase was added. The reverse transcription reaction was carried out at 42°C for 1 h and terminated by heating at 70°C for 5 min. 1 µL of cDNA was used for Real‐time PCR reactions on a CFX384 Touch Real‐Time PCR Detection System (Bio‐Rad Laboratories, Hercules, CA, USA) using the SYBR Green master mix (Applied Biosystems, Foster City, CA, USA). *GAPDH* was used as a housekeeping gene to normalize the data. Primers are listed in Table S1.

### 2.3 Genetic Depletion of YAP and TAZ in hMSCs Using Lentiviral shRNA Transduction

HEK293T cells were transfected with pLV(shYAP1)‐EGFP or pLV(shTAZ)‐mCherry using Lipofectamine 3000 and cultured for 72 h in 100 mm dishes. Viral supernatant was collected, filtered (0.45 μm), and used to transduce hMSCs in the presence of 8 μg/mL polybrene for 24 h. Cells were then switched to fresh DMEM + 10% FBS and maintained with medium changes every 3 days. Transduction efficiency was assessed by EGFP or mCherry fluorescence, and knockdown of YAP or TAZ was confirmed by Western blot.

### 2.4 Western Blot Analysis

Proteins were extracted using RIPA buffer (Cell Signaling Technology) supplemented with protease inhibitors (Roche). Samples were separated by 10% SDS‐PAGE and transferred to PVDF membranes. Membranes were incubated overnight at 4°C with primary antibodies against YAP, p‐YAP, TAZ, p‐TAZ, and HIF1α (1:1000; Cell Signaling Technology) and β‐actin–HRP (1:25,000; Sigma‐Aldrich). After washing, membranes were incubated with HRP‐conjugated secondary antibodies (1:5000) for 2 h at room temperature. Signals were detected by chemiluminescence (Merck Millipore).

### 2.5 ELISA

For ELISA media, cultures were grown for 48 h. Media were harvested, and subjected to IL1‐α and IL6 assays according to the manufacturer's protocol (Biolegend). Briefly, a 96‐well plate (Nunc MaxiSorp) was coated with capture antibody and incubated overnight at 4°C. The plate was then washed four times with PBST before the addition of standards and samples, followed by incubation for 2 h at room temperature (RT) with shaking. After four washes with PBST, the detection antibody was added and incubated for 1 h at RT. Following another four washes, avidin–HRP was added and incubated at RT for 30 min. The plate was then washed five times with PBST, and the signal was developed using TMB substrate for 30 min at RT. The reaction was terminated by adding 2N H_2_SO_4_, and absorbance was measured at 450 nm with background correction at 570 nm.

### 2.6 Conditioned Media Preparation for the HCT116 Cell Proliferation and Endothelial Tube Formation Assay

After cells reached 70%–80%confluence. New culture media was replaced and cultures were grown for 48 h to harvest conditioned media. The harvested conditioned medium was concentrated sixfold (6x) using a 3 kDa Macrosep Advance centrifugal filter (Pall Corporation) by centrifugation at 4000 × g and 4°C for 45 min, or until the desired volume was reached.

### 2.7 HCT116 Cell Proliferation Assay

HCT116 cell proliferation was determined using an xCELLigence system (Roche Diagnostics Deutschland GmbH, Mannheim Germany). HCT116 at 5 × 10^3^ cells were seeded in special gold‐coated 96‐well E‐plates in RPMI1640 supplemented with 2%FBS and incubated overnight to allow cells to attach to the culture plate. On Day 4, half of the culture medium was replaced with fresh testing medium. Cell growth was continuously monitored for a period of 168 h.

### 2.8 Endothelial Tube Formation Assay

HUEHT‐1 cells at 2 × 10^4^ cells were seeded in a 96‐well Matrigel‐coated plate in testing medium diluted with an equal volume of complete medium (EndoGRO, Sigma–Aldrich). After 24 h of culture, the capillary‐like structures derived from HUEHT‐1 cells were photographed and their various characteristic parameters were determined using ImageJ software (Bethesda, MA, USA).

### 2.9 Mycoplasma Test

The medium derived from UE6E7T‐3 culture was periodically collected to test for mycoplasma infection using a MycoAlert PLUS Mycoplasma Detection Kit (Lonza, USA), Table S2.

### 2.10 Ethics Statement

Human chorionic and placental tissues were obtained from healthy donors after written informed consent. All procedures involving human tissues were conducted in accordance with the Declaration of Helsinki and were approved by the Institutional Review Board, Faculty of Medicine Siriraj Hospital, Mahidol University, Bangkok, Thailand (COA No. Si 112/2020; Project: *Role of Hippo Signaling pathway in adult stem/progenitor cells derived from umbilical cord blood and postnatal tissues*). All participants provided written informed consent prior to tissue collection.

### 2.11 Statistical Analysis

All data analyses were performed with the GraphPad Prism software. Data are presented as mean ± standard error of the mean (SEM) of three or more independent experiments. The Kolmogorov ‐Smirnov normality test was used to determine the normal distribution of the groups. An unpaired, two‐tailed Student's *t* test or Mann–Whitney *U* test was used for two‐group comparisons. One‐way ANOVA with Tukey's multiple comparison or Kruskal‐Wallis test was used for multiple comparisons. *p* < 0.05 is considered statistical significance.

## 3 Results

### 3.1 A Three‐Dimensional Culture in Matrigel Enhances the Expression of iCAF Marker Genes, and This Effect Is Further Amplified by Soluble Factors Derived From Cancer Cells

**Figure 1:**
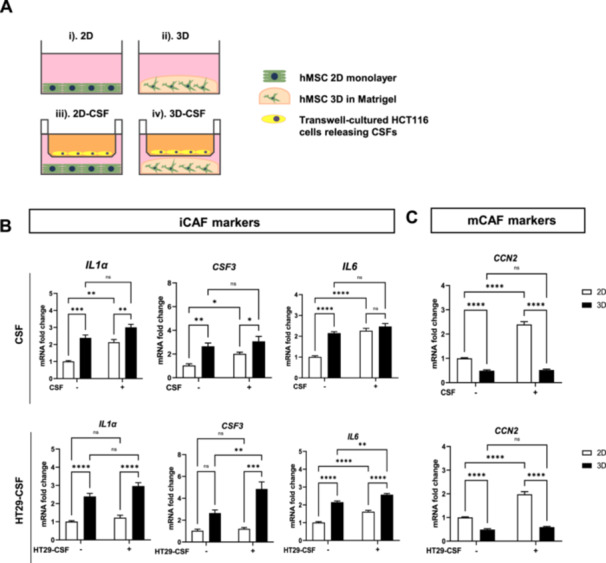
The three‐dimensional culture in Matrigel increases the expression levels of iCAF marker genes in hMSCs. (A) Schematic illustration of the hMSC culture in i) 2D monolayer (2D), ii) 3D culture in Matrigel (3D), iii) 2D in the presence of HCT116‐derived soluble factors through transwell (2D‐CSF), and iv) 3D in the presence of HCT116‐derived soluble factors through transwell (3D‐CSF). (B) Expression levels of iCAF marker genes (*IL1α, CSF3 and IL6*) in BM‐hMSCs culture in 2D, 3D, 2D‐CSF, and 3D‐CSF for 4 days. (C) Expression levels of mCAF markers (*CCN2*) in BM‐hMSCs culture in 2D (white bar) or 3D Matrigel (black bar) and either culture alone or co‐culture with HT29 in trans‐well format (CSF) for 4 days. Data are presented as mean ± SEM of 3 biological replicates. *p < 0.05; **p < 0.01; ***p < 0.001; ****p < 0.0001; ANOVA.

To determine the components of culture conditions that influence the transformation of hMSCs to CAFs, we first studied the effect of various culture conditions on the expression of CAF marker genes by subjecting hMSCs derived from bone marrow (BM‐hMSCs), chorion (CH‐hMSCs) and placenta (PL‐hMSCs) to 4 different culture conditions; two‐dimensional monolayer culture (2D), 2D culture with soluble factors derived from human colon cancer cells, HCT116 (2D‐CSF), three‐dimensional culture in Matrigel (3D), and 3D culture with soluble factors derived from HCT116 cells (3D‐CSF) (Figure 1A). Although the hMSCs in the 2D culture exhibited a typical spindle‐shaped morphology, those subjected to the 3D culture exhibited a more compact shape with multiple cellular processes (Figure S1A). Similar to the hMSCs under 3D culture, the hMSCs under 3D‐CSF condition also exhibited a more compact morphology with multiple cellular processes while those subjected to the 2D‐CSF culture typically retained their spindle‐shaped morphology (Figure S1A; representative images of BM‐hMSCs).

The gene expression study showed that 3D‐culture in Matrigel significantly increased the expression levels of iCAF marker genes (*IL1a, IL6*, and *CSF3*) (Figure 1B) while decreasing the expression levels of the mCAF marker gene, *CCN2* in BM‐hMSCs (Figure 1C). Similar trends were observed in PL‐hMSCs, and CH‐hMSCs compared to those subjected to conventional 2D culture for both iCAF and mCAF genes (Figure S1B,C). Next, we determined the effects of the soluble factor derived from colon cancer cells, HCT116 and HT29, on the expression of iCAF and mCAF marker genes in hMSCs. The results showed that soluble factors derived from HCT116 and HT29 cells further increased the expression levels of iCAF marker genes without affecting the expression level of mCAF marker genes in BM‐hMSCs (Figure 1B,C, bottom row). Similar trends were observed in CH‐hMSCs and PL‐hMSCs Figures S1B,C). In contrast to the hMSCs in 3D culture, soluble factors derived from HCT116 and HT29 cells increased the expression levels of mCAF marker gene, *CCN2*, without altering the expression level of iCAF marker genes in BM‐hMSCs, CH‐hMSCs and PL‐hMSCs subjected to 2D culture (2D‐CSF) compared to those subjected to 2D culture alone (2D) (Figures 1B,C, S1 and S2). These results suggest that 3D culture in Matrigel induces the transformation of hMSCs toward iCAFs, by up‐regulating the expression of iCAF genes while down‐regulating the expression of mCAF genes in these cells. Furthermore, the soluble factors derived from cancer cells enhance the transformation of hMSCs under 3D culture condition to iCAFs by further increasing the expression levels of iCAF marker genes in these cells.

### 3.2 Hypoxic Culture Conditions Further Increased the Transformation of hMSCs to iCAFs

In addition to the matrix and cancer‐derived soluble factors, the hypoxic condition is also an important part of the tumor microenvironment and could therefore affect the transformation of hMSCs to CAFs. To investigate this, BM‐hMSCs, PL‐hMSCs, and CH‐hMSCs were subjected to 3D‐CSF culture, which has been shown to increase iCAF marker expression, under hypoxic condition and analyzed for the expression of iCAF marker genes compared to those under normoxic condition.

**Figure 2:**
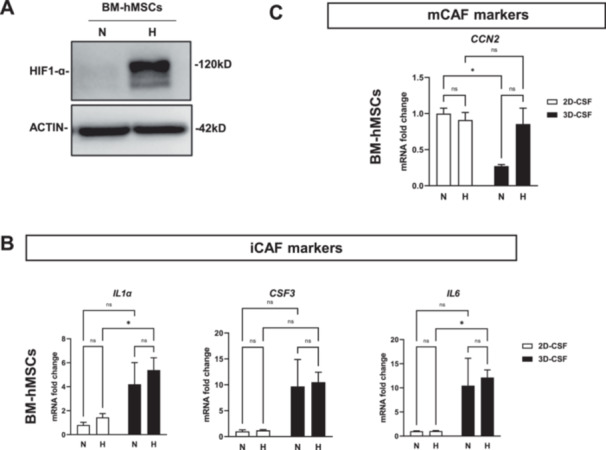
Hypoxia enhances the effect of colon cancer–derived soluble factors on iCAF gene induction in hMSCs cultured in 3D. (A) Western blot of the hypoxic marker HIF1‐α in BM‐hMSCs embedded in 3D Matrigel and co‐cultured with HCT116 via transwell for 4 days under normoxia (20% O_2_, N) or hypoxia (5% O_2_, H). (B) qRT‐PCR analysis of iCAF markers (*IL1α, CSF3, IL6*) in BM‐hMSCs treated with HCT116‐derived soluble factors under 2D (2D‐CSF, white) or 3D (3D‐CSF, black) culture for 4 days in normoxia or hypoxia. (C) Expression levels of mCAF markers (*CCN2*) in BM‐hMSCs culture in 2D (2D‐CSF, white) or 3D (3D‐CSF, black) culture for 4 days in normoxia or hypoxia. Data are mean ± SEM (*n* = 3). *p < 0.05; ns; ANOVA.

As expected, the BM‐hMSCs, CH‐hMSCs and PL‐hMSCs subjected to 3D‐CSF culture under hypoxic (H) condition showed an increased level of hypoxia inducible factor 1‐α (HIF1‐α), a master regulator of hypoxic response, compared with those cultured under normoxic condition (Figures 2A and S3A). While, the hypoxic condition (H) did not significantly promote expression of iCAF and mCAF marker genes in BM‐hMSCs (Figure 2B,C), but further increased the expression of iCAF marker genes without affecting the expression level of mCAF marker genes in CH‐hMSCs and PL‐hMSCs subjected to 3D‐CSF condition compared to their counterparts cultured under normoxic condition (N) (Figure 2B,C). Unlike hMSCs subjected to 3D‐CSF culture, the hypoxic condition (H) did not increase the expression levels of any iCAF marker genes in BM‐hMSCs, CH‐hMSCs and PL‐hMSCs subjected to 2D‐CSF culture compared to their counterparts cultured under normoxic condition (N) (Figure 2B and S3B).

The positive effect of the hypoxic condition on the expression of iCAF marker genes in PL‐hMSCs subjected to the 3D‐CSF condition was further confirmed by the levels of IL1a and IL6 proteins secreted from these cells. The result showed that the PL‐hMSCs subjected to 3D‐CSF culture under hypoxic condition secreted significantly higher levels of IL1a and IL6 proteins compared to their counterparts cultured under normoxic condition (Figure S3D). These results clearly demonstrated that a combination of 3D culture, cancer‐derived soluble factors, and hypoxic condition synergistically induce the transformation of hMSCs to iCAFs.

### 3.3 A Combination of Three‐Dimensional Culture in Matrigel, Cancer‐Derived Soluble Factors, and Hypoxia Transforms Tumor‐Suppressive hMSCs Into Tumor‐Promoting CAFs

**Figure 3:**
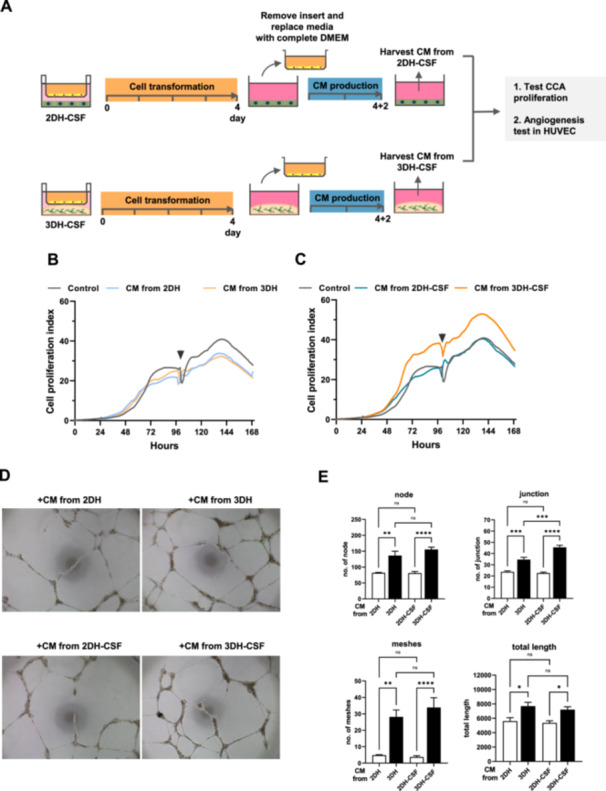
Combined 3D culture, cancer‐derived factors, and hypoxia convert tumor‐suppressive hMSCs into tumor‐promoting CAFs. (A) Schematic of conditioned media (CM) collected from BM‐hMSCs cultured in 2D or 3D and co‐cultured with HCT116 under hypoxia (2DH‐CSF vs. 3DH‐CSF). (B) Growth kinetics of HCT116 cells treated with CM from BM‐hMSCs cultured in 2D (2DH, light blue) or 3D (3DH, light orange) under hypoxia. DMEM + 10% FBS served as control (gray). (C) Growth kinetics of HCT116 cells treated with CM from BM‐hMSCs co‐cultured with HCT116 in 2D (2DH‐CSF, dark blue) or 3D (3DH‐CSF, dark orange) under hypoxia. (D) Representative images of capillary‐like structures formed by HUVECs treated with CM from 2DH, 3DH, 2DH‐CSF, and 3DH‐CSF conditions. (E) Quantification of tube formation parameters in HUVECs treated with the indicated CM. Data are mean ± SEM (*n* = 8). *P < 0.05; **P < 0.01; ***P < 0.001; ****P < *0.0001*; unpaired *t* test.

Next, we determined whether 3D culture, cancer‐derived soluble factors, hypoxic condition, or a combination of these factors can change the effects of hMSCs on cancer cell proliferation and angiogenesis. To investigate this, we subjected BM‐hMSCs to 4 different conditions which are (i) 2D culture under hypoxic conditions (2DH), (ii) 3D culture under hypoxic conditions (3DH), (iii) 2D‐CSF under hypoxic condition (2DH‐CSF), and (iv) 3D‐CSF under hypoxic condition (3DH‐CSF). After cultured under these conditions for 4 days, the conditioned media (CM) were collected to test their effects on the proliferation of HCT116 cells and the vessel formation capacity of human endothelial vein endothelial cells (HUVECs) (Figure 3A).

The results showed that the soluble factors derived from the cultured hMSCs under 2DH and 3DH conditions suppressed the proliferation of HCT116 cells compared to the control (DMEM + 10% FBS) (Figure 3B). There is no discernible difference in the level of suppressive effect on HCT116 proliferation between soluble factors derived from hMSCs subjected to 2DH and 3DH conditions (Figure 3B).

Interestingly, when 2DH‐ and 3DH‐hMSCs were treated with cancer‐derived soluble factors (CSF), the effect of their released soluble factors changed dramatically. Unlike 2DH‐ and 3DH‐hMSCs, the soluble factors derived from the 2DH‐CSF and 3DH‐CSF hMSCs no longer suppressed the proliferation of HCT116 cells compared to the control (DMEM + 10% FBS) (Figure 3C). In case of 3DH‐CSF hMSCs, CSF treatment even reversed the effect of their released soluble factors from inhibiting to promoting HCT116 proliferation compared to controls (Figure 3C). Unlike 3DH‐CSF hMSCs, although CSF treatment abolished the suppressive effect of 2DH‐hMSC‐derived soluble factors on HCT116 proliferation, it did not reverse the effect from inhibiting to promoting HCT116 proliferation compared to controls (Figure 3C).

Regarding angiogenesis, the conditioned medium derived from 3DH‐hMSCs increased the vessel formation capacity of HUVECs, determined by an increase in the number of nodes, junctions, meshes and the total length of the capillary‐like structure derived from HUVECs compared to those of 2DH hMSCs (Figure 3D,E). Unlike the effect on HCT116 proliferation, CSF treatment of 2DH‐ and 3DH‐hMSCs did not further increase the effect of their soluble factors in promoting the vessel formation capacity of HUVECs (Figure 3D,E).

These results suggest that while a combination of 3D culture and hypoxic condition (3DH) is sufficient to increase the ability of hMSCs to promote angiogenesis, the addition of cancer‐derived soluble factors to this particular condition (3DH‐CSF) is essential to transform tumor‐suppressing hMSCs into tumor‐promoting CAFs.

### 3.4 A Combination of Three‐Dimensional Culture, Cancer‐Derived Soluble Factors, and Hypoxia Reduced YAP and TAZ Levels in hMSCs

YAP and TAZ, the main effectors of the Hippo signaling pathway, have been suggested to play a role in the induction of CAF phenotypes in some cancer stromal cells. Therefore, we investigated whether a combination of 3D culture, cancer‐derived soluble factors, and hypoxia, which transform tumor‐suppressing hMSCs into tumor‐promoting CAFs, affects the levels of YAP and TAZ proteins in these cells.

**Figure 4:**
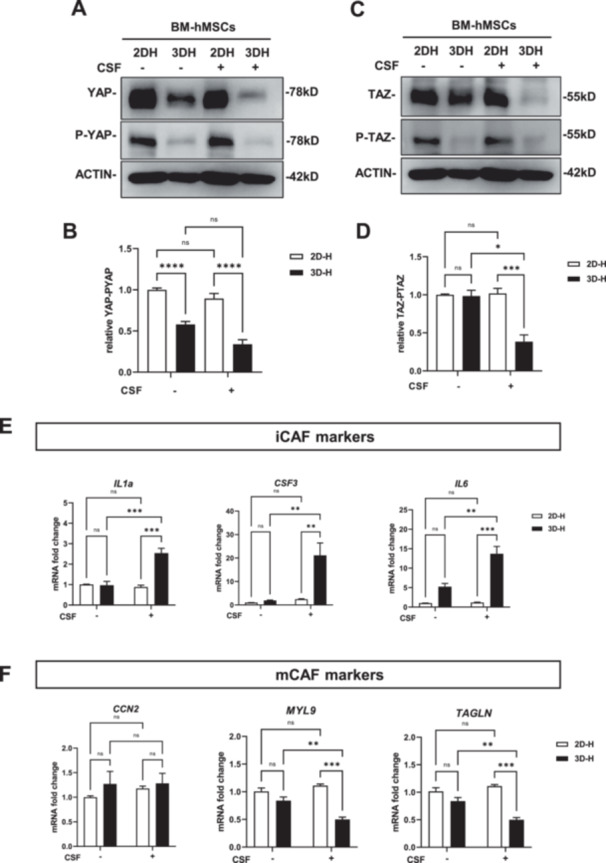
3D culture, cancer‐derived soluble factors, and hypoxia reduce YAP/TAZ levels in hMSCs. (A) Representative western blots showing YAP and P‐YAP in BM‐hMSCs cultured under 2DH, 3DH, 2DH‐CSF, or 3DH‐CSF conditions for 4 days. ACTIN serves as loading control. (B) Quantification of YAP and P‐YAP levels under the indicated conditions. (C) Representative western blots of TAZ and P‐TAZ in BM‐hMSCs subjected to 2DH, 3DH, 2DH‐CSF, and 3DH‐CSF. (D) Quantification of TAZ and P‐TAZ levels. Data are mean ± SEM (*n* = 3). *P < *0.05*; ***P < *0.001*; ANOVA. (E) qRT‐PCR analysis of iCAF markers (*IL1α, CSF3, IL6*) and (F) mCAF markers (*CCN2, MYL9, TAGLN*) in BM‐hMSCs after 4 days in 2DH, 3DH, 2DH‐CSF, or 3DH‐CSF conditions. Data are mean ± SEM (*n* = 3). **P < 0.01; ***P < *0.001;* ANOVA. The ACTIN loading control shown in both Figure 4A,C corresponds to the same ACTIN blot from the membrane used for phosphorylated YAP and phosphorylated TAZ detection. The original ACTIN blots from both the total and phosphorylated protein membranes are provided in Figures S9 and S10.

BM‐hMSCs were subjected to a 2D or 3D culture under hypoxic condition (H) and treated with soluble factors derived from HCT116 cells (CSF). The cells were then harvested to determine the levels of YAP and TAZ proteins. The results showed that 3D culture under hypoxic condition (3DH) significantly decreased YAP and TAZ levels, as well as the YAP/p‐YAP and TAZ/pTAZ ratios in BM‐hMSCs compared to those subjected to 2D culture under the same condition (2DH) (Figure 4A–D). Furthermore, CSF treatment further decreased YAP and TAZ levels, as well as the YAP/p‐YAP and TAZ/pTAZ ratios in 3DH‐hMSCs (3DH‐CSF) compared to those without CSF treatment (3DH) (Figure 4A–D).

Similar to BM‐hMSCs, the 3DH condition also significantly decreased YAP and TAZ levels, as well as the YAP/p‐YAP and TAZ/pTAZ ratios in PL‐hMSCs and CH‐hMSCs compared to those subjected to the 2DH condition (Figure S4). Also similar to BM‐hMSCs, CSF treatment further decreased YAP and TAZ levels, as well as the YAP/p‐YAP and TAZ/pTAZ ratios in 3DH‐hMSCs (3DH‐CSF) compared to those without CSF treatment (3DH) (Figure S4). These results suggest that a combination of 3D culture, cancer‐derived soluble factors, and hypoxic condition reduced YAP and TAZ levels in hMSCs, possibly by decreasing the production and increasing the phosphorylation of these proteins.

Next, we determined whether the down‐regulation of YAP and TAZ observed in hMSCs induced by a combination of 3D culture, cancer‐derived soluble factors, and hypoxia is associated with their increased expression of iCAF markers. Indeed, the results showed that downregulation of YAP and TAZ upregulated iCAF gene expression (Figure 4E), down‐regulated mCAF gene expression (Figure 4F), and increased the levels of secreted IL1α and IL6 in hMSCs (Figure S5).

### 3.5 Down‐Regulation of YAP and TAZ Increases the Expression Levels of the iCAF Marker Without Significantly Affecting the Expression Levels of the mCAF Marker in hMSCs

**Figure 5:**
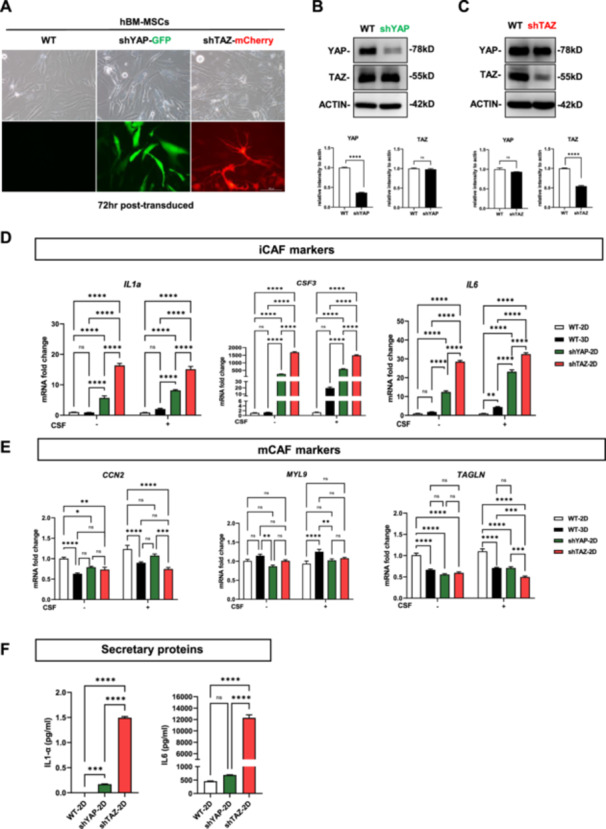
YAP/TAZ downregulation promotes iCAF marker expression without significantly affecting mCAF marker expression in hMSCs. (A) Bright‐field and fluorescent images of BM‐hMSCs 72 h after transduction with pLenti‐shYAP‐GFP or pLenti‐shTAZ‐mCherry. Scale bar = 100 µm. (B) Western blots and quantification of YAP and TAZ levels in shYAP‐hMSCs versus WT controls. (C) Western blots and quantification of YAP and TAZ levels in shTAZ‐hMSCs versus WT controls. Data are mean ± SEM (*n* = 3). ****P < *0.0001;* unpaired t test. (D) qRT‐PCR analysis of iCAF markers (*IL1α, CSF3, IL6*) and (E) mCAF markers (*CCN2, MYL9, TAGLN*) in shYAP‐ and shTAZ‐hMSCs under 2D and 2D‐CSF conditions, compared with WT hMSCs under 2D, 3D, 2D‐CSF, and 3D‐CSF conditions. Data are mean ± SEM (*n* = 3). *P < *0.05*; **P < 0.01; ***P < 0.001; ****P < *0.0001*; ANOVA. (F) Levels of IL1α and IL6 secreted from shYAP‐ and shTAZ‐hMSCs under 2D culture, compared with WT cells. Data are mean ± SEM (*n* = 3). ***P < 0.001; ****P < *0.0001*; ANOVA.

To further confirm that YAP and TAZ play a crucial role in regulating the expression of iCAF and mCAF markers, we reduced the expression levels of YAP and TAZ in BM‐hMSC using shRNA against the *YAP* or *TAZ* genes (Figure 5A). As expected, western blot analysis confirms a significant reduction in YAP and TAZ levels in these cells (Figure 5B,C).

Next, we determined the expression levels of the iCAF and mCAF marker genes in these YAP or TAZ targeted BM‐hMSCs (shYAP‐BM‐hMSCs and shTAZ‐BM‐hMSCs). The results showed that reducing the levels of YAP or TAZ greatly increased the expression levels of all iCAF marker genes (*IL1a, CSF3*, and *IL6*) (Figure 5D) without significantly affecting the expression of mCAF marker genes (*CCN2* and *MYL9*) (Figure 5E). The expression levels of the *IL1a, CSF3* and *IL6* genes in shYAP‐BM‐hMSCs and shTAZ‐BM‐hMSCs subjected to a conventional 2D culture under normoxic conditions were even higher than those of wild‐type BM‐hMSCs subjected to a combination of 3D culture, HCT116‐CM, and hypoxia (3D‐CSF WT) (Figure 5D,E). It should be noted that the expression levels of the *IL1a, CSF3*, and *IL6* genes in shTAZ‐hMSCs were significantly higher than those of shYAP‐hMSCs cultured under the same conditions (Figure 5D). Similar to the results observed in BM‐hMSCs, reducing the levels of YAP or TAZ generally increase the expression levels of iCAF without significantly affecting the expression levels of most mCAF markers in PL‐hMSCs (Figure S6) and CH‐hMSCs (Figure S7).

Consistent with iCAF gene expression, reduction of YAP or TAZ levels in BM‐hMSCs subjected to a conventional 2D culture significantly increased the levels of secreted IL1a and IL6 proteins in the medium derived from these cells compared to the wild‐type control (Figure 5F). Again, the levels of secreted IL1a and IL6 proteins in the medium derived from shTAZ‐hMSCs were significantly greater than the medium derived from shYAP‐hMSCs cultured under the same condition (Figure 5F), suggesting that TAZ might play a more prominent role than YAP in this process. These results suggest that down‐regulation of YAP and TAZ in hMSCs induced by a combination of 3D culture, cancer‐derived soluble factors, and hypoxia could be responsible for the increased expression of the iCAF marker in these cells.

### 3.6 Down‐Regulation of YAP and TAZ Is Sufficient to Transform hMSCs Into Tumor‐Promoting CAFs

**Figure 6:**
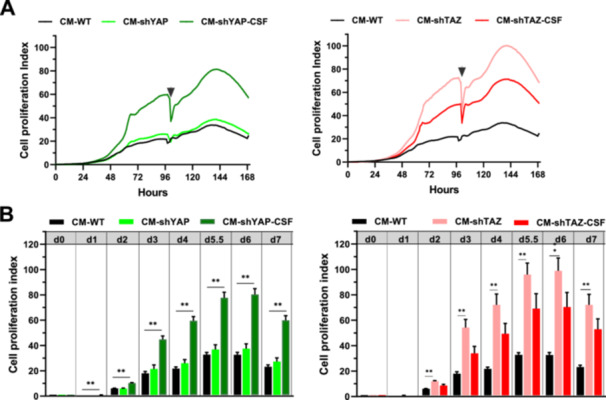
YAP/TAZ downregulation converts hMSCs into tumor‐promoting CAFs. (A) Growth kinetics of HCT116 cells treated with conditioned media (CM) from shYAP‐ or shTAZ‐transduced BM‐hMSCs (CM‐shYAP, CM‐shTAZ) under 2D culture, compared with CM from WT hMSCs (CM‐WT) and with HCT116‐derived soluble factors (CSF). The arrowhead indicates the time of medium replacement. Data represent mean of 3 biological replicates. (B) Day‐by‐day analysis of HCT116 growth under the same conditions. Data are mean ± SEM (*n* = 3). **P < *0.01*; ANOVA.

Next, we determined whether down‐regulation of YAP and TAZ in hMSCs is sufficient to transform these cells into tumor‐promoting CAFs. To investigate this, we collected CM from shYAP‐BM‐hMSCs (CM‐shYAP) and shTAZ‐BM‐hMSCs (CM‐shTAZ) and tested their effects on HCT116 proliferation concurrently with the CM from the wild‐type counterpart (CM‐WT). Consistent with the gene expression study described above, soluble factors derived from shTAZ‐BM‐hMSCs significantly increased the proliferation of HCT116 cells compared to the control (HCT116 cultured in DMEM + 10% FBS) while soluble factors derived from their wild‐type counterparts did not have this effect (Figure 6A,B). Although the soluble factors derived from shYAP‐BM‐hMSC‐derived soluble factors (CM‐shYAP) did not increase the proliferation of HCT116 cells compared to the control CM‐WT, pre‐treating these shYAP‐hMSCs with CSF (CM‐shYAP‐CSF) greatly increased their positive effect on HCT116 cell proliferation (Figure 6A,B).

## 4 Discussion

The findings of this study provide significant insight into the role of the tumor microenvironment, particularly the three‐dimensional (3D) culture, cancer‐derived soluble factors and hypoxic conditions, in driving the transformation of human mesenchymal stem cells (hMSCs) into cancer‐associated fibroblasts (CAFs). Specifically, we observed that a combination of these conditions significantly increases the expression of iCAF (inflammatory CAF) marker genes, while reducing the expression of mCAF (myofibroblast CAF) marker genes in hMSCs.

Our data show that 3D culture in Matrigel significantly increases the expression of iCAF marker genes (*IL1a, CSF3, IL6*) in BM‐hMSCs, CH‐hMSCs and PL‐hMSCs compared to conventional 2D culture. This aligns with previous reports demonstrating that 3D culture better mimics the in vivo environment, promoting more complex interactions and cellular behaviors compared to 2D culture (Habanjar et al. 2021; Humpel 2015). Up‐regulation of iCAF markers under these conditions suggests that the spatial architecture and extracellular matrix (ECM) of the 3D system could induce an iCAF‐like phenotype in hMSCs. This is consistent with a previous report showing that 2D culture promotes CAFs to exhibit a myofibroblast phenotype (myCAF), while 3D tumor spheroid cultures exhibit a more inflammatory phenotype (iCAF) (Yang et al. 2024). This underscores the role of 3D culture in the regulation of CAF behavior and phenotype by potentially mimicking the tumor microenvironment in vivo (Brewer et al. 2025).

Incorporation of the second component, soluble factors derived from HCT116 colon cancer cells, into 3D cultures further increases the expression of iCAF marker genes and proteins in all hMSCs examined. On the contrary, colon cancer‐derived soluble factors do not increase the expression of iCAF markers in BM‐hMSCs, PL‐hMSCs, and CH‐hMSCs in 2D cultures. These results suggest that the soluble factors released by cancer cells play a crucial role in inducing the iCAF phenotype and that 3D culture makes hMSCs more permissive to this transformation process by sensitizing these cells to tumor‐derived signals.

In addition to 3D culture and colon cancer cell‐derived soluble factors, our results show that hypoxic conditions significantly enhance the expression of iCAF marker genes and increases the secretion of IL1a and IL6, pro‐inflammatory cytokines typically associated with iCAFs. These findings are consistent with the previous studies showing that hypoxia, which is prevalent in tumor microenvironment, regulates gene expression and metabolic adaptation of both cancer and cancer stromal cells leading to the cancer progression (Capik and Karatas 2025; Gilkes et al. 2014; Guo et al. 2022). Similarly to the colon cancer‐derived soluble factors, the effect of hypoxia on increasing the expression of the iCAF marker in PL‐hMSCs and CH‐hMSCs in 2D cultures was much lower than that of those subjected to 3D culture. These results suggest that 3D culture also makes hMSCs more permissive to hypoxic‐induced iCAF transformation. Transformation of hMSCs to iCAFs was confirmed by our functional assays showing that soluble factors derived from the transformed MSCs increase colon cancer cell proliferation and endothelial angiogenesis, while soluble factors derived from their non‐transformed counterparts suppress colon cancer cell proliferation. It should be noted that the effect of hypoxia on the induction of the expression of the iCAF marker in BM‐hMSCs under 3D culture was less than those of PL‐hMSCs and CH‐hMSCs under the same culture condition. This difference can be attributed to the physiologic hypoxic microenvironment within bone marrow tissue, where BM‐MSCs reside, making these cells less responsive to hypoxic condition during our CAF transformation culture.

The Hippo signaling pathway and its downstream effectors, YAP and TAZ, has been implicated in the regulation of CAF phenotypes in various cancer stromal cells (Humpel 2015; Yang et al. 2024). Consistent with this, our results show that a combination of 3D culture, cancer‐derived soluble factors, and hypoxia not only downregulated the levels of YAP and TAZ proteins in hMSCs, but also increases the phosphorylation and inactivation of these proteins. These results suggest that a combination of 3D culture, cancer‐derived soluble factors, and hypoxia could transform hMSCs into the iCAF phenotype by down‐regulating the expression of YAP and TAZ in these cells. The roles of YAP and TAZ in the transformation of hMSCs to iCAFs were confirmed by our shRNA knockdown experiments. Reduced expression of YAP and TAZ in hMSC in 2D culture, which previously did not induce the iCAF phenotype, led to a significant increase in the expression of iCAF markers (*IL1a, IL6, CSF3*) without significantly affecting the expression of most mCAF markers (*CCN2* and *MYL9*) in these cells. Consistent with the expression of the iCAF marker, soluble factors derived from TAZ knockdown hMSCs significantly increase the proliferation of HCT116 cells compared to their wild‐type counterpart. Although soluble factors derived from YAP knockdown hMSCs did not increase HCT116 cell proliferation, pre‐treating these cells with cancer‐derived soluble factors greatly increases their proliferation‐promoting effect on HCT116 cells. The different effect on proliferation enhancement between TAZ knockdown and YAP knockdown hMSCs could be explained by a different level of iCAF gene expression between these two cells (Figure 6B). While TAZ knockdown hMSCs, which already express very high levels of iCAF genes, can increase HCT116 proliferation by themselves, YAP knockdown hMSCs, whose expression of iCAF genes is more modest, might require cancer‐derived soluble factors to further increase their iCAF gene expression to levels sufficient to promote HCT116 cell proliferation.

Taken together, our findings suggest that a combination of 3D culture, cancer‐derived soluble factors, and hypoxia collectively induce the transformation of hMSCs into tumor‐promoting iCAFs through the down‐regulation of YAP and TAZ levels in these cells. Furthermore, reducing YAP and TAZ levels in hMSCs is not only sufficient to increase the expression of iCAF markers, but can transform these cells into tumor‐promoting iCAFs. The critical role of YAP and TAZ in this transformation, highlights their potential as therapeutic targets for developing new treatments by modulating the CAF phenotype in the cancer stroma.

## 5 Conclusions

This study reveals a novel mechanism by which 3D culture, cancer‐derived soluble factors, and hypoxia collectively induce the transformation of hMSCs into tumor‐promoting iCAFs through downregulation of YAP and TAZ, offering potential therapeutic avenues for developing new treatment by modulating the CAF phenotype in the cancer stroma. However, additional studies are necessary to investigate the molecular mechanisms underlying YAP and TAZ down‐regulation in the tumor microenvironment and the role of other signaling pathways, such as NF‐kB or STAT3, in the regulation of the iCAF phenotype to provide better insight into CAF biology and its role in tumor progression.

## Author Contributions

**Pimjai Chingsuwanrote:** data curation, investigation, methodology, writing – original draft, validation. **Chanchao Lorthongpanich:** conceptualization, formal analysis, funding acquisition, writing – review and editing, writing – original draft, software, visualization. **Pakpoom Kheolamai:** conceptualization, formal analysis, writing – review and editing, validation, project administration. **Ting Gang Chew:** resources, supervision, writing – review and editing. **Chuti Laowtammathron:** writing – review and editing, resources, supervision. **Sudjit Luanpitpong:** writing – review and editing, resources, supervision. **Rangsun Parnpai:** resources, supervision, writing – review and editing. **Surapol Issaragrisil:** supervision, resources, writing – review and editing.

## Consent

Written informed consent was obtained from all participants for the collection and use of chorionic and placental tissues in this study.

## Conflicts of Interest

The authors declare no conflicts of interest.

## Supporting information


**Figure S1:** (associated with Figure 1). The three‐dimensional culture in Matrigel decreased the expression levels of mCAF marker genes in hMSCs. (A) Representative images of primary CH‐hMSCs cultured in 2D and 3D with and without CSF for 4 days. Scale bar = 100 µm. Arrowhead indicates cell with compact shape with multiple cellular processes cultured in 3D. (B) qPCR analysis of iCAF markers (*IL1α, CSF3, IL6*) and (C) mCAF markers (*CCN2*) in CH‐hMSCs and PC‐hMSCs culture in 2D (white bar) or 3D Matrigel (black bar). Results show mean ± SEM of 3 biological replicates. ****p < 0.0001. ANOVA.
**Figure S2:** (associated with Figure 1). Co‐culturing with HT29 show similar result as to decreased the expression levels of mCAF marker genes in CH‐ and PL‐hMSCs. (A) qPCR analysis of iCAF markers (*IL1α, CSF3, IL6*) and mCAF markers (*CCN2*) in CH‐hMSCs and (B) in PC‐hMSCs culture in 2D (white bar) or 3D Matrigel (black bar) co‐culture with HT29 in trans‐well format (CSF) for 4 days. Results show mean ± SEM of 3 biological replicates. ****p < 0.0001. ANOVA.
**Figure S3:** (associated with Figure 2). Hypoxia enhances iCAF gene induction in hMSCs cultured in 3D. (A) Western blot of the hypoxic marker HIF1‐α in CH‐ and PL‐hMSCs embedded in 3D Matrigel and co‐cultured with HCT116 via transwell for 4 days under normoxia (20% O_2_, N) or hypoxia (5% O_2_, H). (B) qRT‐PCR analysis of iCAF markers (*IL1α, CSF3, IL6*) in CH‐ and PL‐hMSCs treated with HCT116‐derived soluble factors under 2D (2D‐CSF, white) or 3D (3D‐CSF, black) culture for 4 days in normoxia or hypoxia. (C) Expression levels of mCAF markers (CCN2) in BM‐hMSCs culture in 2D (2D‐CSF, white) or 3D (3D‐CSF, black) culture for 4 days in normoxia or hypoxia. Data are mean ± SEM (*n* = 3). *p < 0.05; ***p < 0.001; ****p < 0.0001; ns; ANOVA. (D) Levels of IL1α and IL6 secreted from PL‐hMSCs cultured in 3D with HCT116 under normoxia or hypoxia. Data are presented as mean ± SEM of 3 biological replicates. *P < 0.05, ns = not significant difference. Unpaired Student *t* test.
**Figure S4:** (associated with figure 4). A combination of three‐dimensional culture, cancer‐derived soluble factors, and hypoxia reduced YAP and TAZ levels in hMSCs. (A) Western blot analysis of YAP and P‐YAP (left) and TAZ and P‐TAZ (right) in PL‐hMSCs in 2D or 3D Matrigel with or without co‐culture with HCT116 in trans‐well format, all conditions were incubated under hypoxia for 4 days. Representative images and quantitative analysis from 3 biological replicates. Loading control, ACTIN. Results show mean ± SEM from 3 biological replicates. ***P < 0.001; ****P < 0.0001. ANOVA. (B) Western blot analysis of YAP and P‐YAP(left) and TAZ and P‐TAZ(right) in CH‐hMSCs in 2D or 3D Matrigel with or without co‐culture with HCT116 in trans‐well format, all conditions were incubated under hypoxia for 4 days. Representative images and quantitative analysis from 3 biological replicates. Loading control, ACTIN. Results show mean ± SEM from 3 biological replicates. ****P < 0.0001. ANOVA. The same ACTIN loading control is shown in both the left and right panels of (A), as it was obtained from the membrane used for total YAP and total TAZ detection. Similarly, the same ACTIN loading control is shown in both the left and right panels of (B), as it was obtained from the membrane used for phosphorylated YAP and phosphorylated TAZ detection. The original ACTIN blots from both the total and phosphorylated protein membranes are provided in Supplementary Figures 13–16.
**Figure S5:** (associated with figure 4). A combination of three‐dimensional culture, cancer‐derived soluble factors, and hypoxia reduced YAP and TAZ levels in hMSCs. (A) ELISA of IL1α and IL6 from supernatant of BM‐hMSCs culture in 2D (white bar) or 3D Matrigel (black bar) and either culture alone or co‐culture with HCT116 in trans‐well format under hypoxia condition for 4 days. Results show mean ± SEM of 3 biological replicates. *P < 0.05; ***P < 0.001; ****p < 0.0001. ANOVA. (B) qPCR analysis of iCAF markers (*IL1α, CSF3 and IL6*) and mCAF markers (*CCN2*, *MYL9* and *TAGLN*) from BM‐hMSCs in 3D Matrigel and co‐culture with HCT116 in trans‐well format under normoxic (white bar) and hypoxic conditions (black bar). Results show mean ± SEM of 3 biological replicates. ANOVA.
**Figure S6:** (associated with Figure 5). The effects of YAP/TAZ downregulation on the expression levels of iCAF and mCAF marker genes in PL‐hMSCs. (A) Bright‐field and fluorescent images of PL‐hMSCs 72 h after transduction with pLenti‐shYAP‐GFP or pLenti‐shTAZ‐mCherry. Scale bar = 100 µm. (B) Western blots and quantification of YAP and TAZ levels in shYAP‐PL‐hMSCs vs. WT controls. *P < 0.05; **P < 0.01; ***P < 0.001; ****P < 0.0001; unpaired *t* test. (C) Western blots and quantification of YAP and TAZ levels in shTAZ‐PL‐hMSCs vs. WT controls. Data are mean ± SEM (n = 3). *P < 0.05; **P < 0.01; ***P < 0.001; ****P < 0.0001; unpaired *t* test. (D) qRT‐PCR analysis of iCAF markers (*IL1α, CSF3, IL6*) and mCAF markers (*CCN2, MYL9, TAGLN*) in shYAP‐ and shTAZ‐PL‐hMSCs under 2D and 2D‐CSF conditions, compared with WT PL‐hMSCs under 2D, 3D, 2D‐CSF, and 3D‐CSF conditions. Data are mean ± SEM (n = 3). *P < 0.05; **P < 0.01; ***P < 0.001; ****P < 0.0001; ANOVA.
**Figure S7:** (associated with Figure 5). The effects of YAP/TAZ downregulation on the expression levels of iCAF and mCAF marker genes in CH‐hMSCs. (A) Bright‐field and fluorescent images of CH‐hMSCs 72 h after transduction with pLenti‐shYAP‐GFP or pLenti‐shTAZ‐mCherry. Scale bar = 100 µm. (B) Western blots and quantification of YAP and TAZ levels in shYAP‐CH‐hMSCs vs. WT controls. *P < 0.05; **P < 0.01; ***P < 0.001; ****P < *0.0001;* unpaired *t* test. (C) Western blots and quantification of YAP and TAZ levels in shTAZ‐CH‐hMSCs vs. WT controls. Data are mean ± SEM (n = 3). *P < 0.05; **P < 0.01; ***P < 0.001; ****P < 0.0001; unpaired *t* test. (D) qRT‐PCR analysis of iCAF markers (*IL1α, CSF3, IL6*) and mCAF markers (*CCN2, MYL9, TAGLN*) in shYAP‐ and shTAZ‐CH‐hMSCs under 2D and 2D‐CSF conditions, compared with WT CH‐hMSCs under 2D, 3D, 2D‐CSF, and 3D‐CSF conditions. Data are mean ± SEM (n = 3). *P < 0.05; **P < 0.01; ***P < 0.001; ****P < *0.0001;* ANOVA.
**Figure S8:** (associated with Figure 2A). Full‐uncropped gels from Western blot analysis associated with the Figure 2A. The selected band of each protein is represented in red box.
**Figure S9:** (associated with figure 4A). Full‐uncropped gels from Western blot analysis associated with the figure 4 A. The selected band of each protein is represented in red box. The black box indicates original ACTIN blot from the same membrane as the YAP blot shown in Figure 4 A. The samples loaded onto all membranes were derived from the same experiment.
**Figure S10:** (associated with figure 4C). Full‐uncropped gels from Western blot analysis associated with the figure 4 C. The selected band of each protein is represented in red box. The black box indicates original ACTIN blot from the same membrane as the TAZ blot shown in Figure 4 C. The samples loaded onto all membranes were derived from the same experiment.
**Figure S11:** (associated with Figure 5B). Full‐uncropped gels from Western blot analysis associated with the Figure 5B. The selected band of each protein is represented in red box.
**Figure 12:** (associated with Figure 5C). Full‐uncropped gels from Western blot analysis associated with the Figure 5C. The selected band of each protein is represented in red box.
**Figure S13:** (associated with supplement figure 4A left). Full‐uncropped gels from Western blot analysis associated with the supplement figure 4 A (left). The selected band of each protein is represented in red box. The black box indicates original ACTIN blot from the same membrane as the P‐YAP blot shown in Figure 4 A (left panel). The samples loaded onto all membranes were derived from the same experiment.
**Figure S14:** (associated with supplement figure 4A right). Full‐uncropped gels from Western blot analysis associated with the supplement figure 4 A (right). The selected band of each protein is represented in red box. The black box indicates original ACTIN blot from the same membrane as the P‐TAZ blot shown in Figure 4 A (right panel). The samples loaded onto all membranes were derived from the same experiment.
**Figure S15:** (associated with supplement figure 4B left). Full‐uncropped gels from Western blot analysis associated with the supplement figure 4B (left). The selected band of each protein is represented in red box. The black box indicates original ACTIN blot from the same membrane as the YAP blot shown in Figure 4B (left panel). Samples loaded to all membranes were from the same experiment. The samples loaded onto all membranes were derived from the same experiment.
**Figure S16:** (associated with supplement figure 4B right). Full‐uncropped gels from Western blot analysis associated with the supplement figure 4B (right). The selected band of each protein is represented in red box. The black box indicates original ACTIN blot from the same membrane as the YAP blot shown in Figure 4B (right panel). The samples loaded onto all membranes were derived from the same experiment.

**Table S1:** Primers used for quantitative real‐time polymerase chain reaction.

**Table S2:** Mycoplasma test result of uE6E7T‐3.

## Data Availability

The data that supports the findings of this study are available in the supplementary information of this article.
