# Supplementary material for: Hippo Pathway Regulation and Microenvironmental Cues Governing Human MSC Transition to Cancer‐Associated Fibroblasts
Source: J Cell Physiol. 2026 Aug 5;241(8):e70219. doi: 10.1002/jcp.70219 (PMC13440140; doi:10.1002/jcp.70219)
Supplement: Supplementary file 1 [file JCP-241-0-s002.pdf]

Supplementary Figure 1

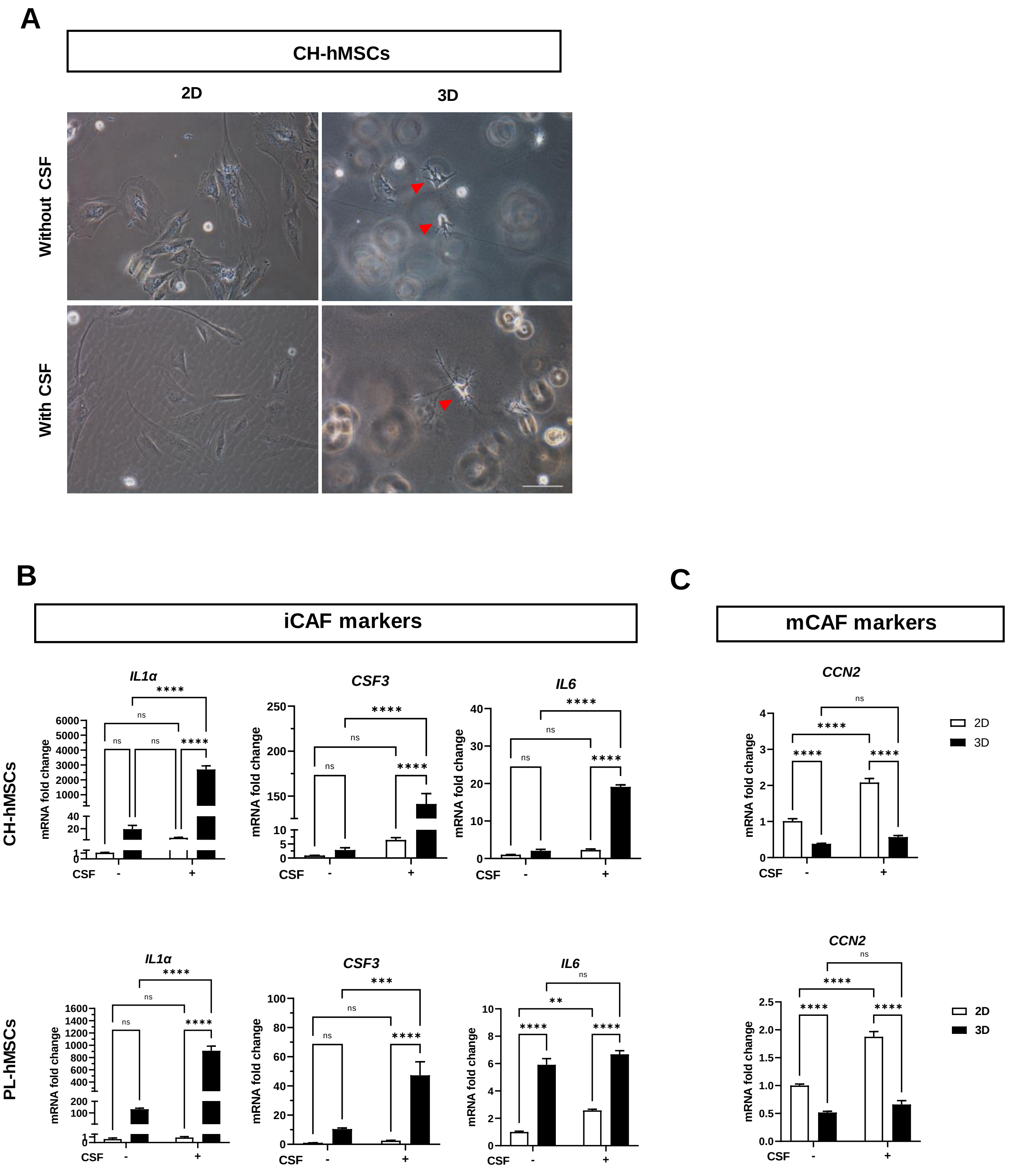

Supplementary Figure 2

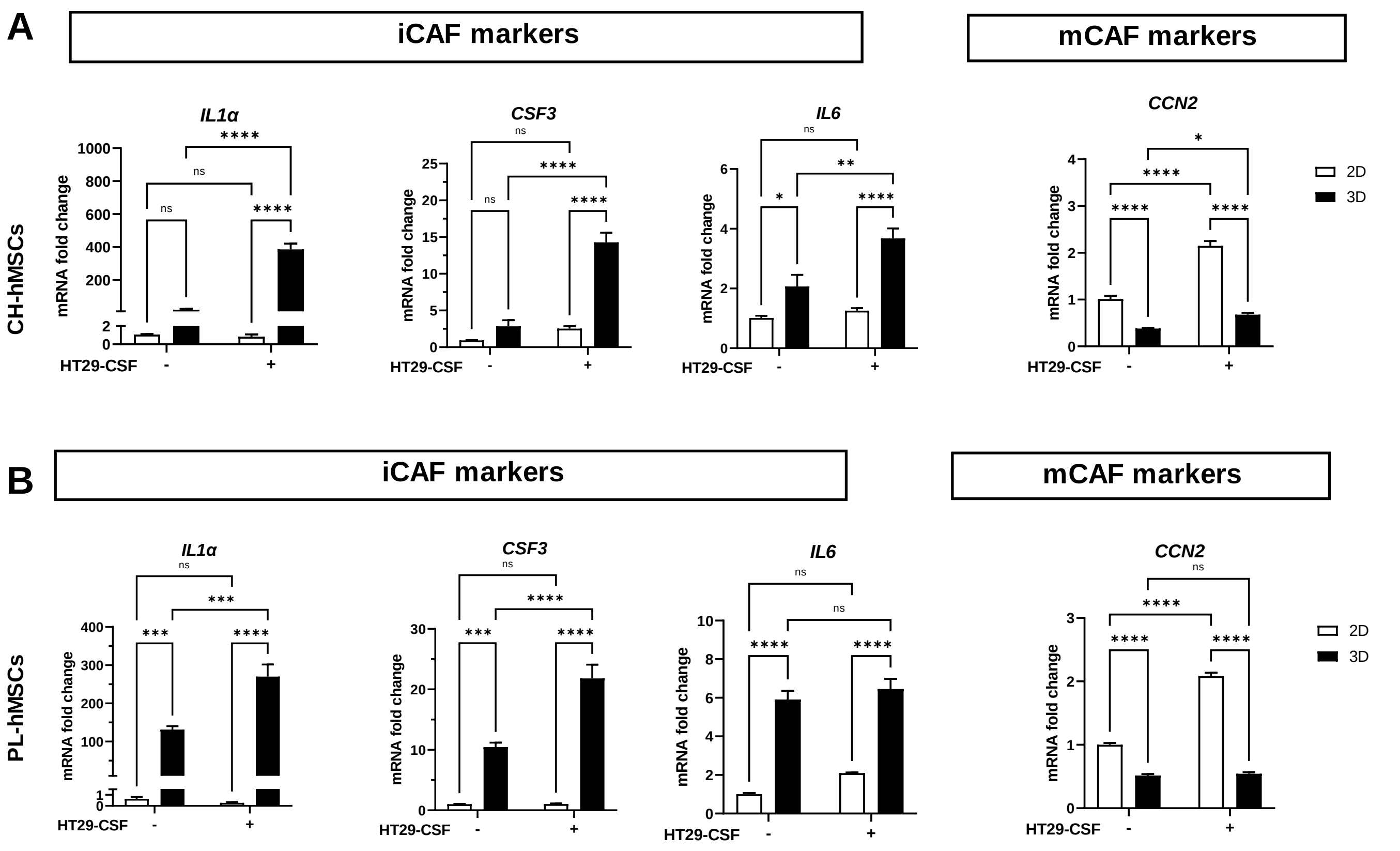

Supplementary Figure 3

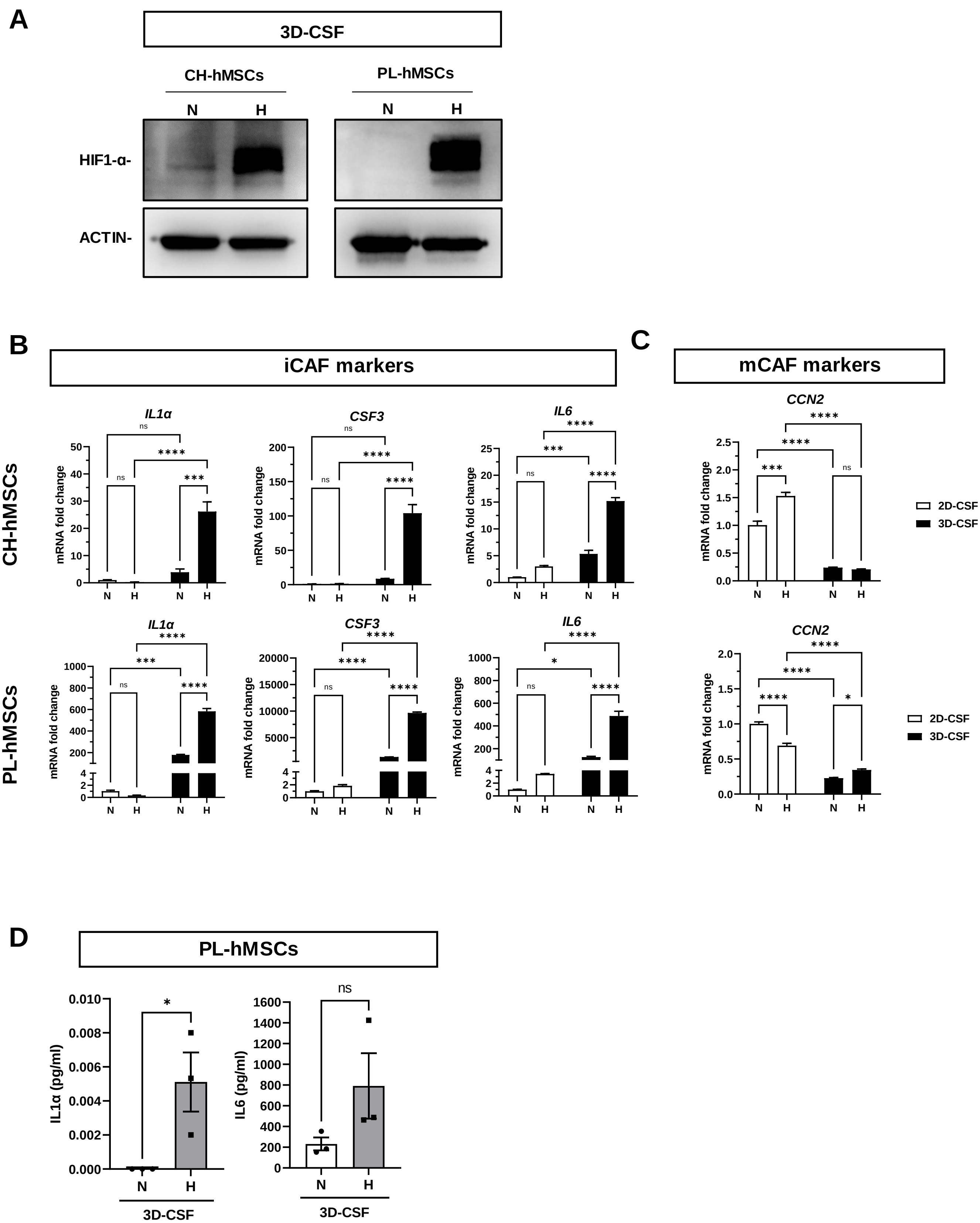

Supplementary Figure 4

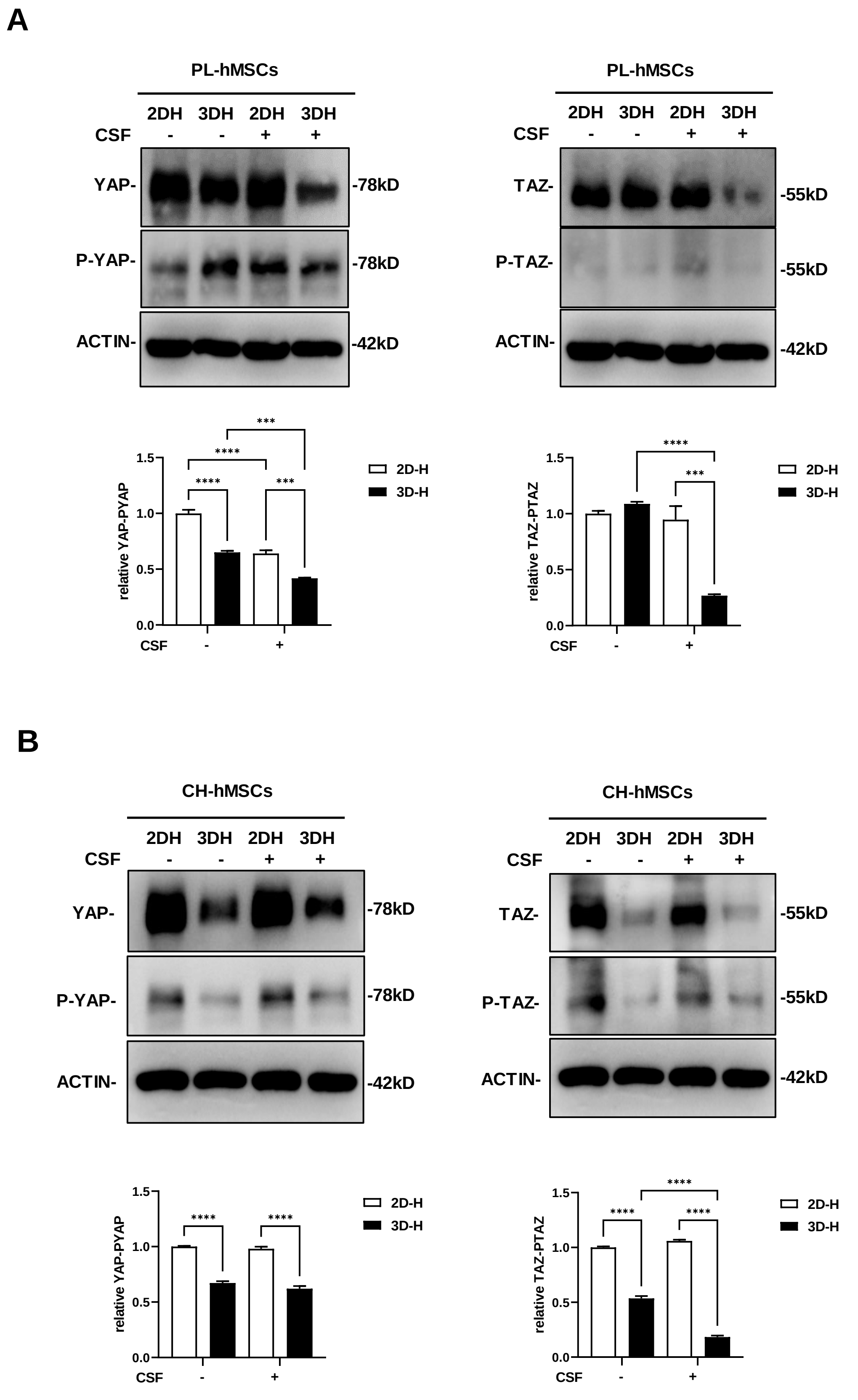

Supplementary Figure 5

A

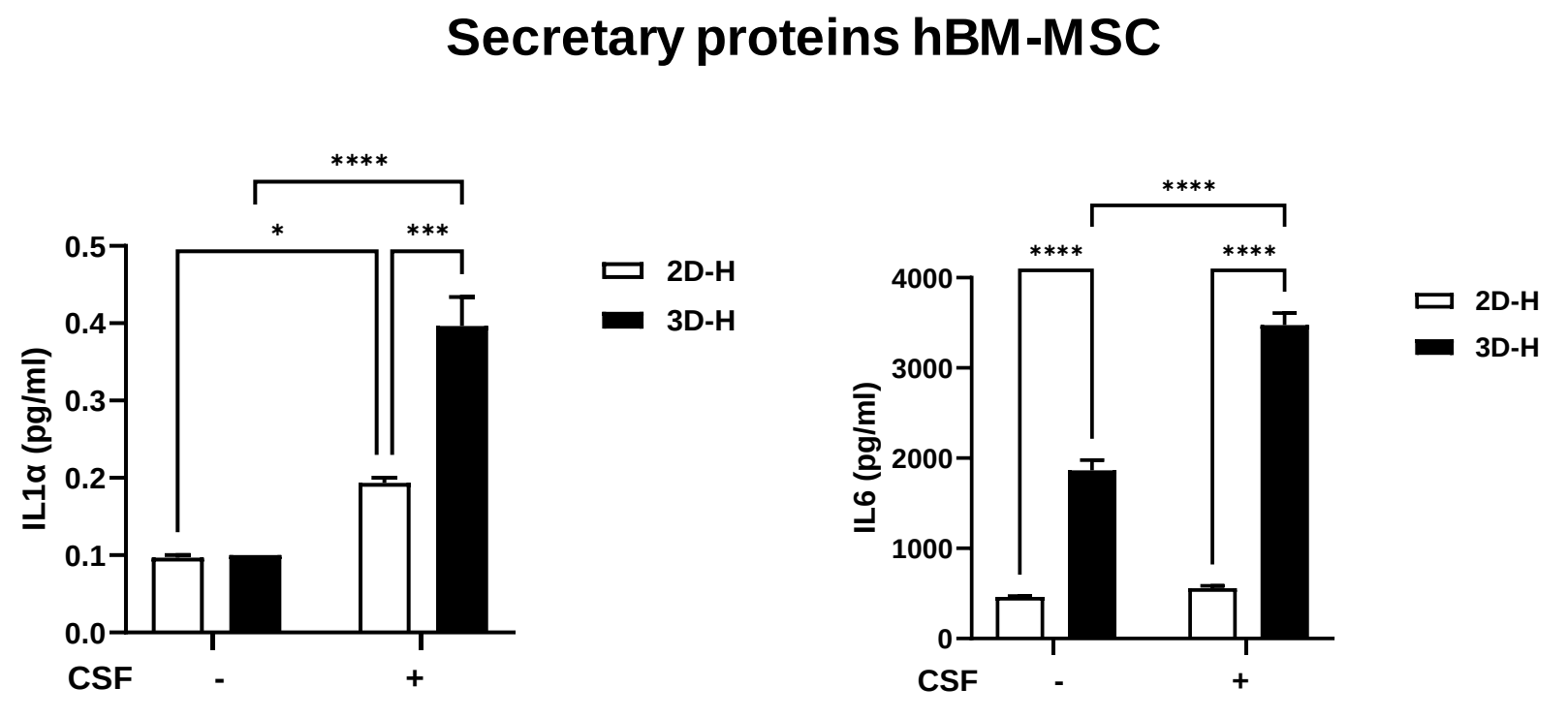

B

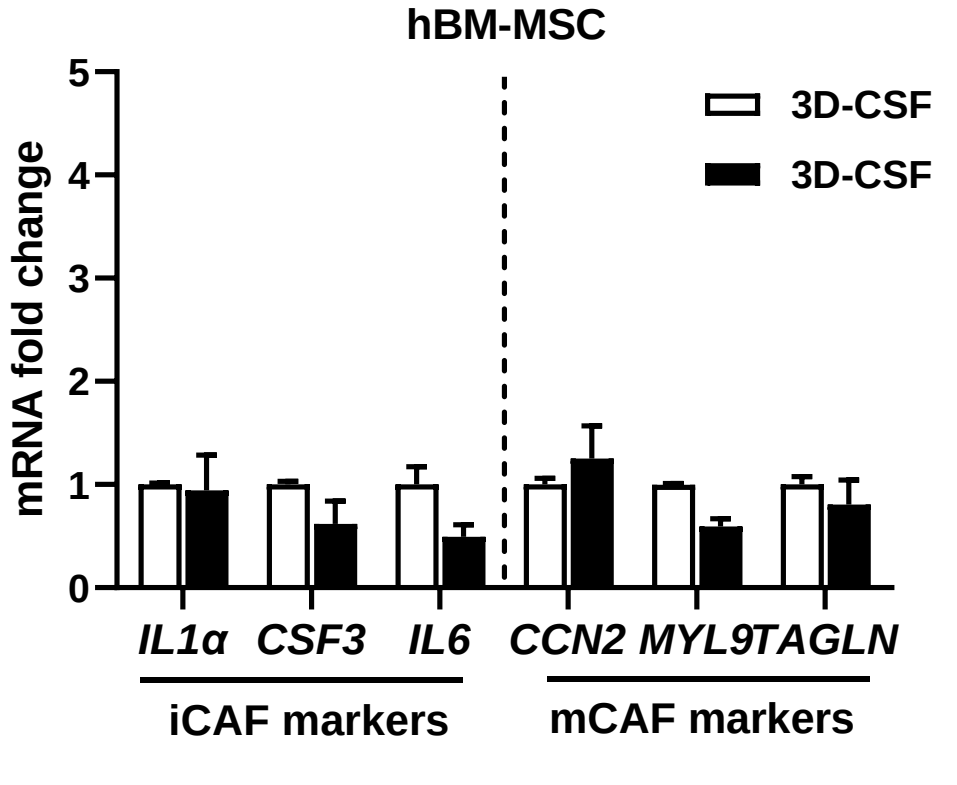

Supplementary Figure 6

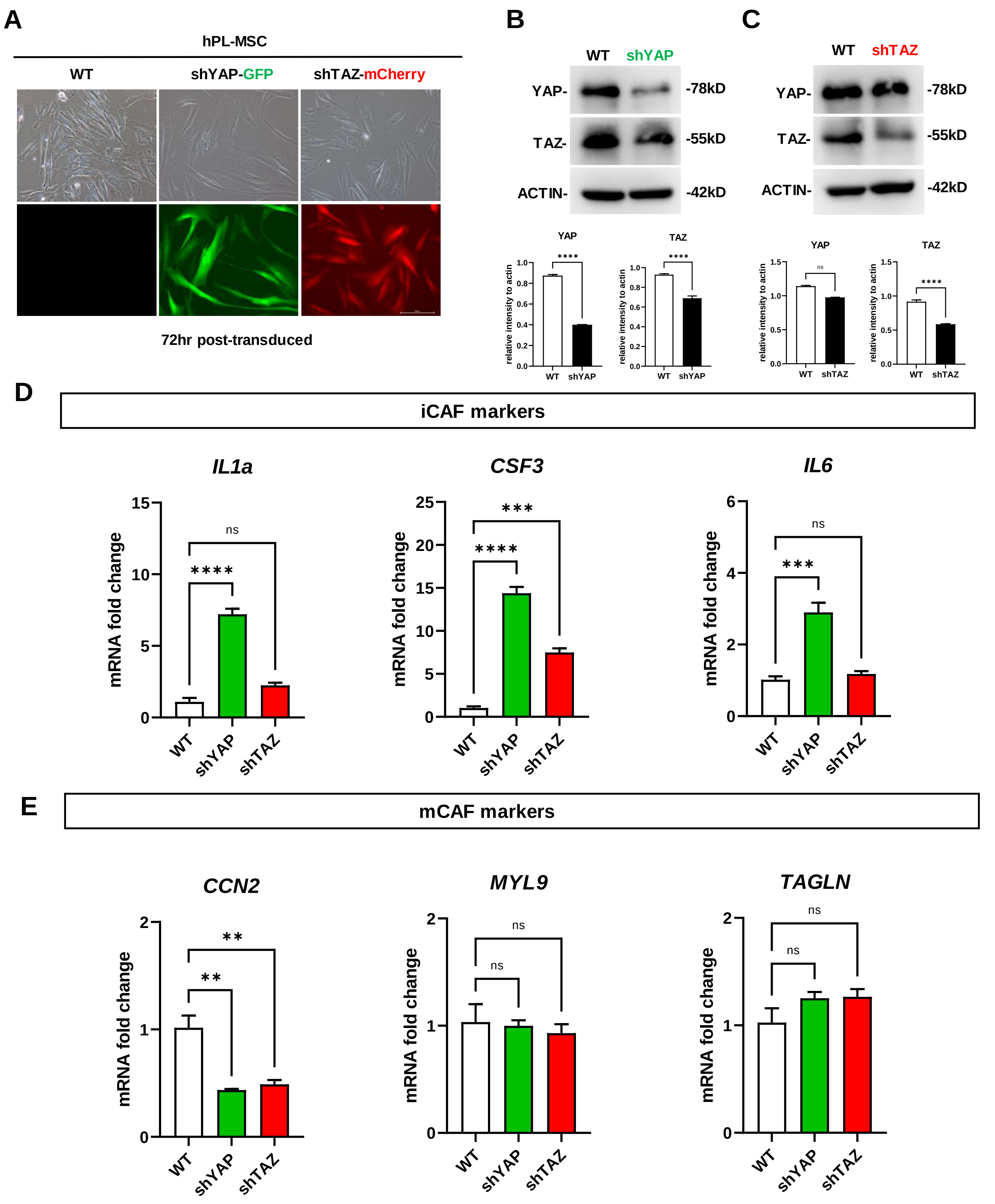

Supplementary Figure 7

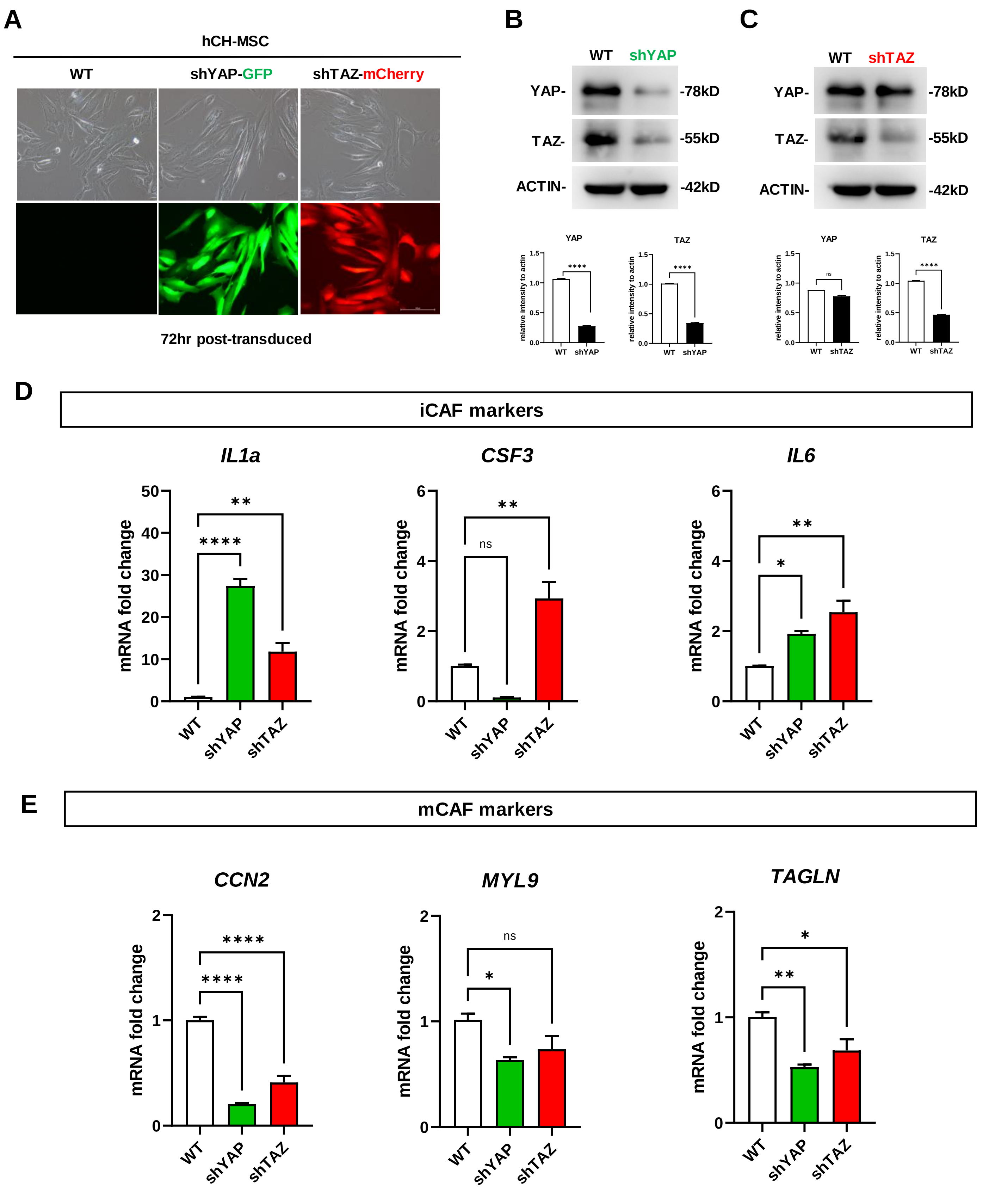

Supplementary Figure 8
Associated with Figure 2A

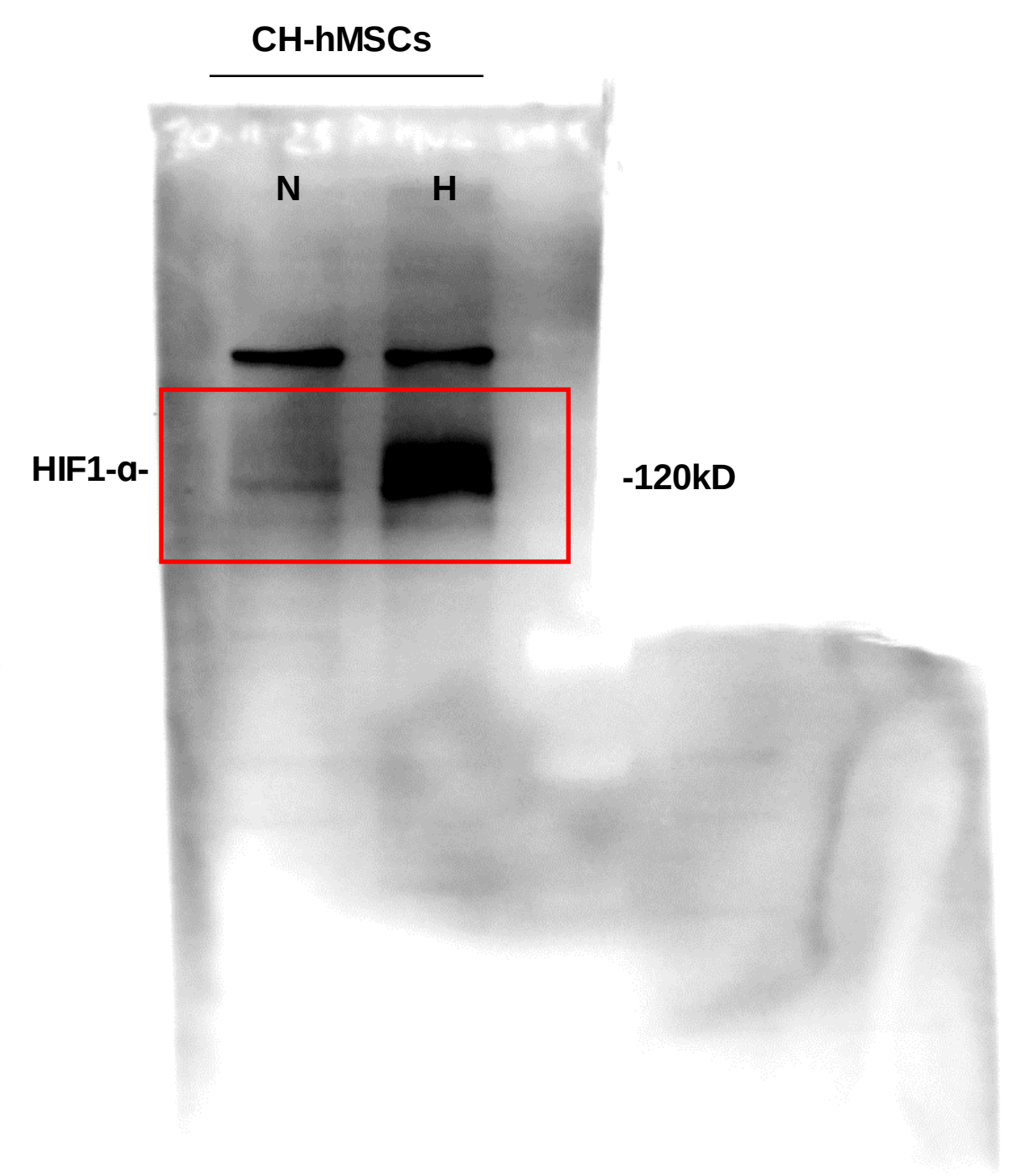

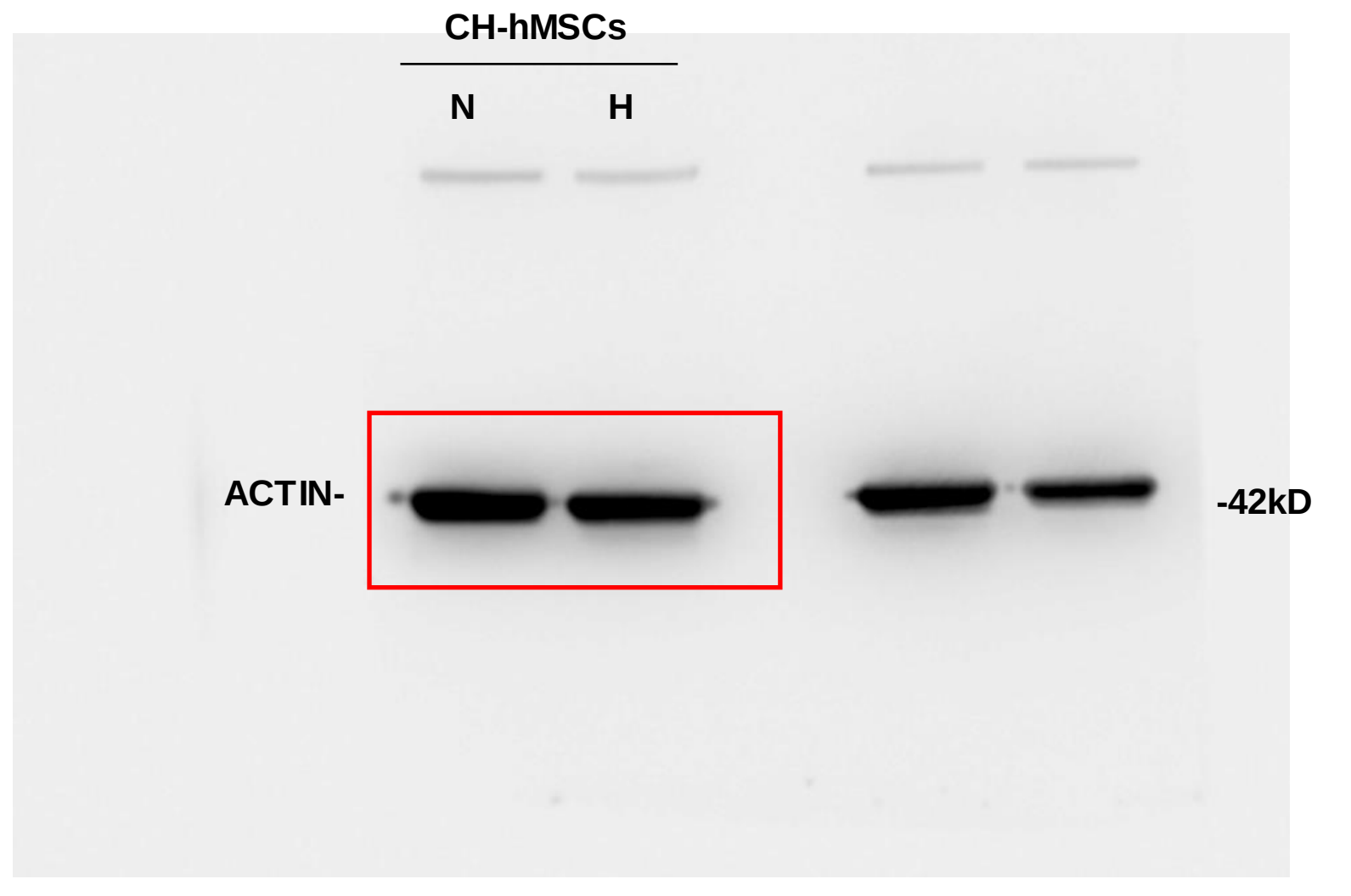

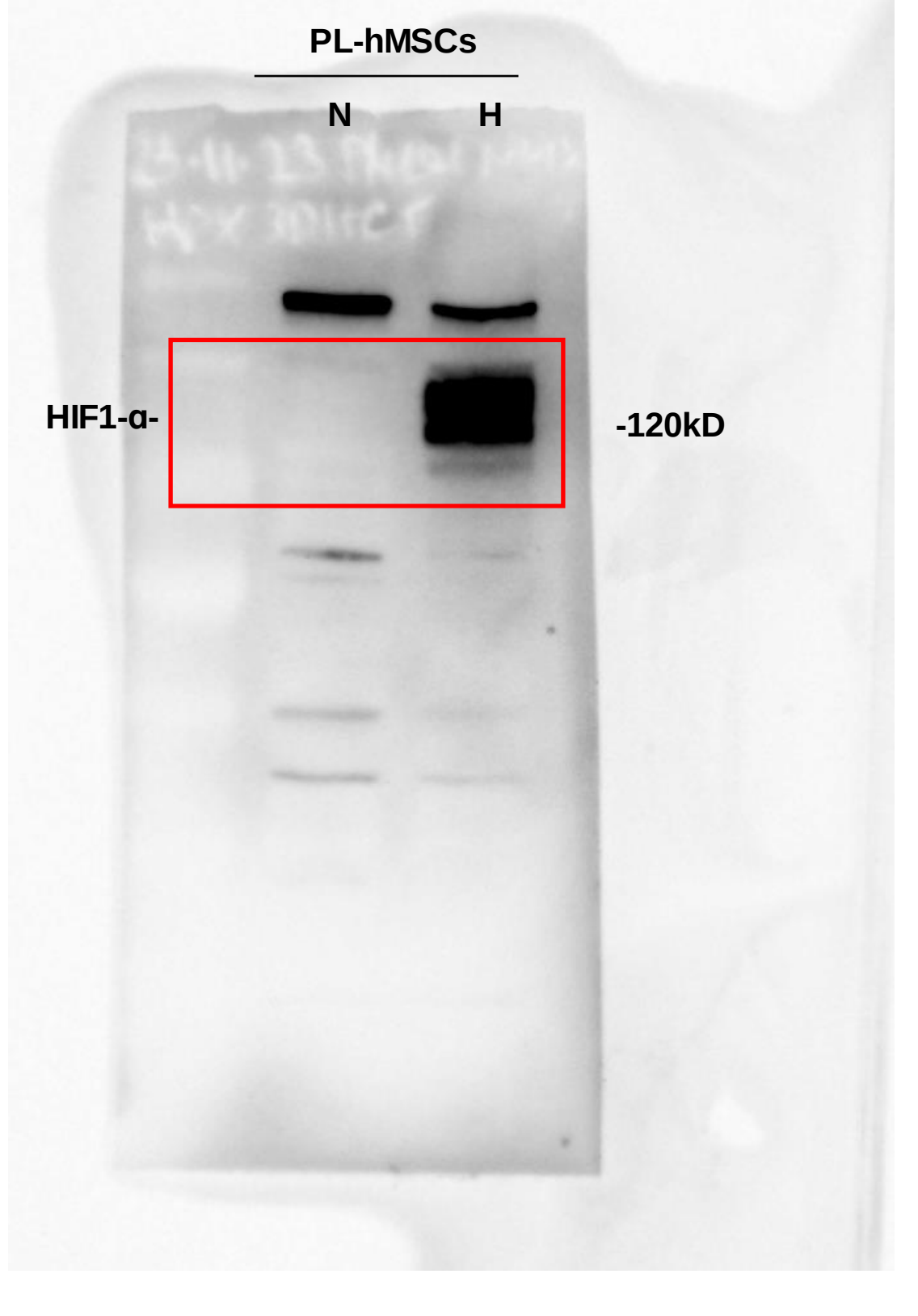

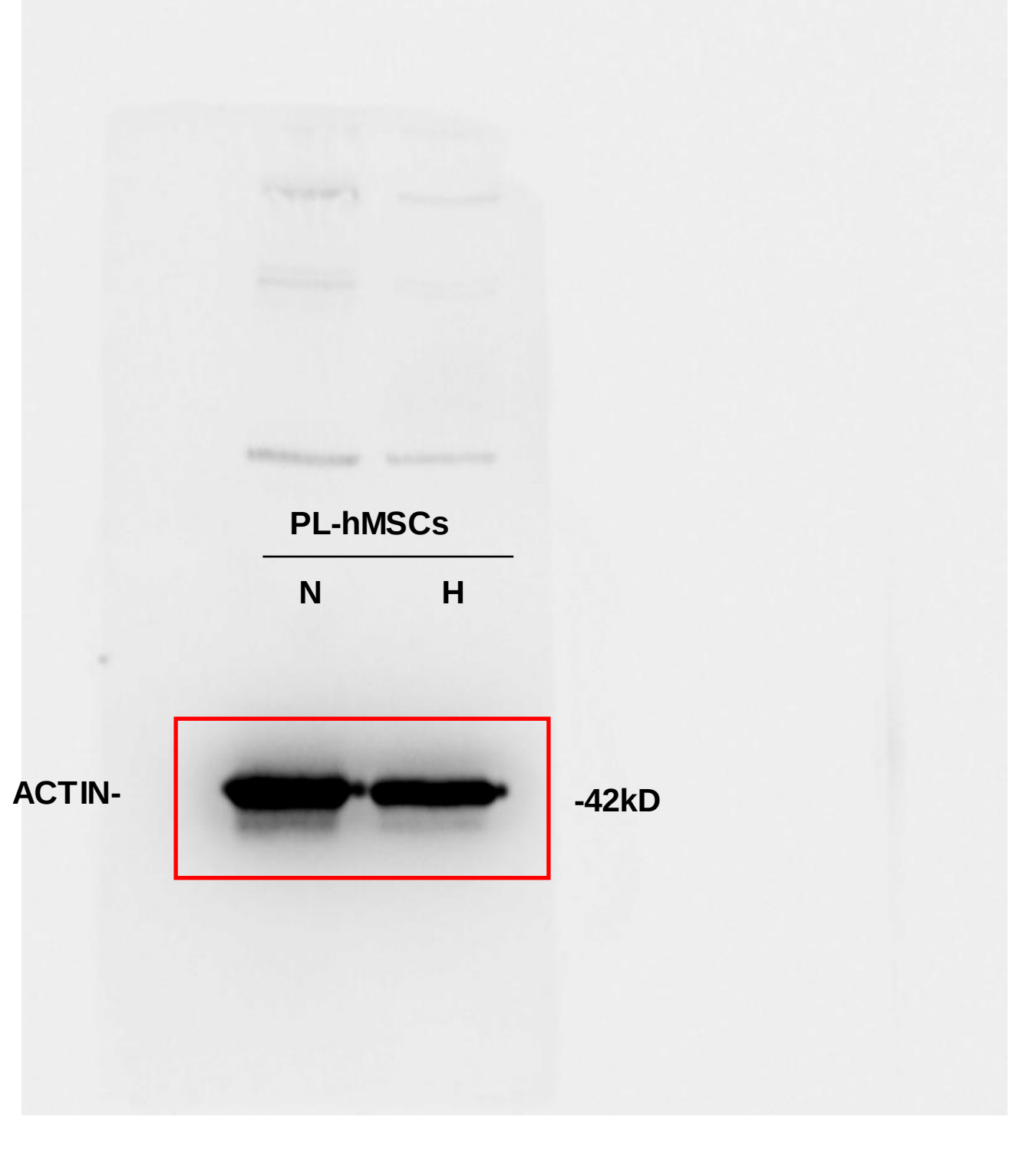

Supplementary Figure 8 (continue)
Associated with Figure 2A

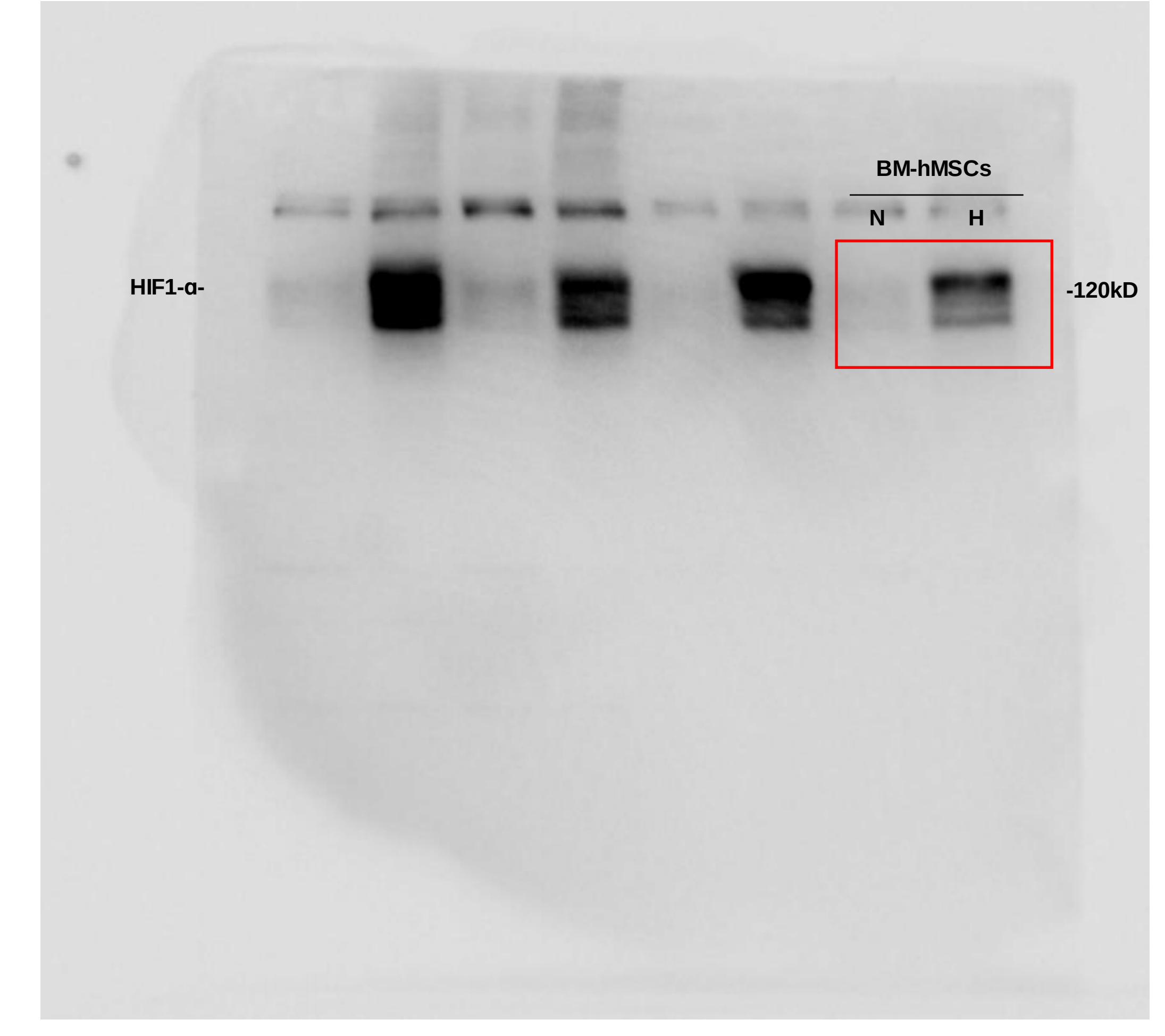

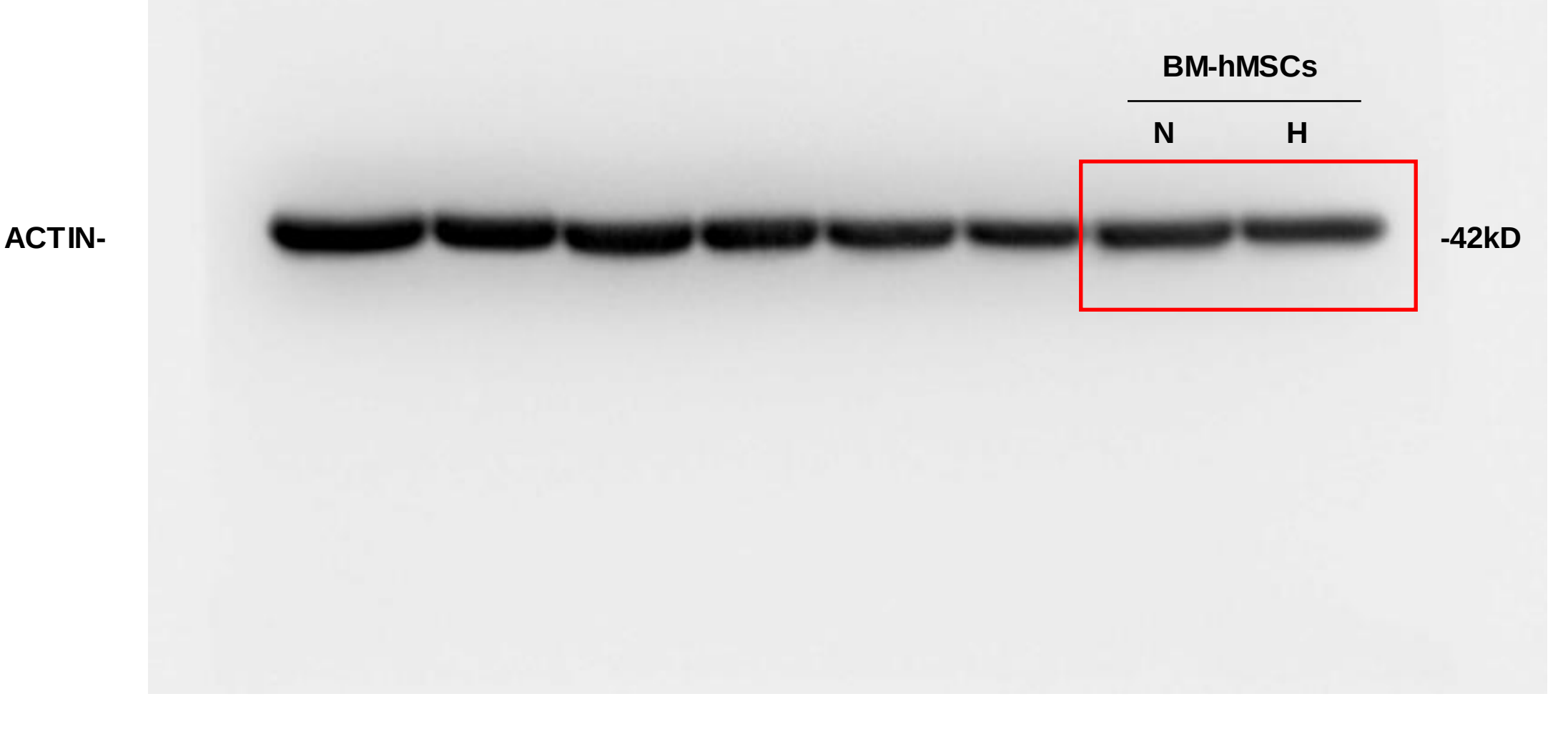

Supplementary Figure 9
Associated with Figure 4A

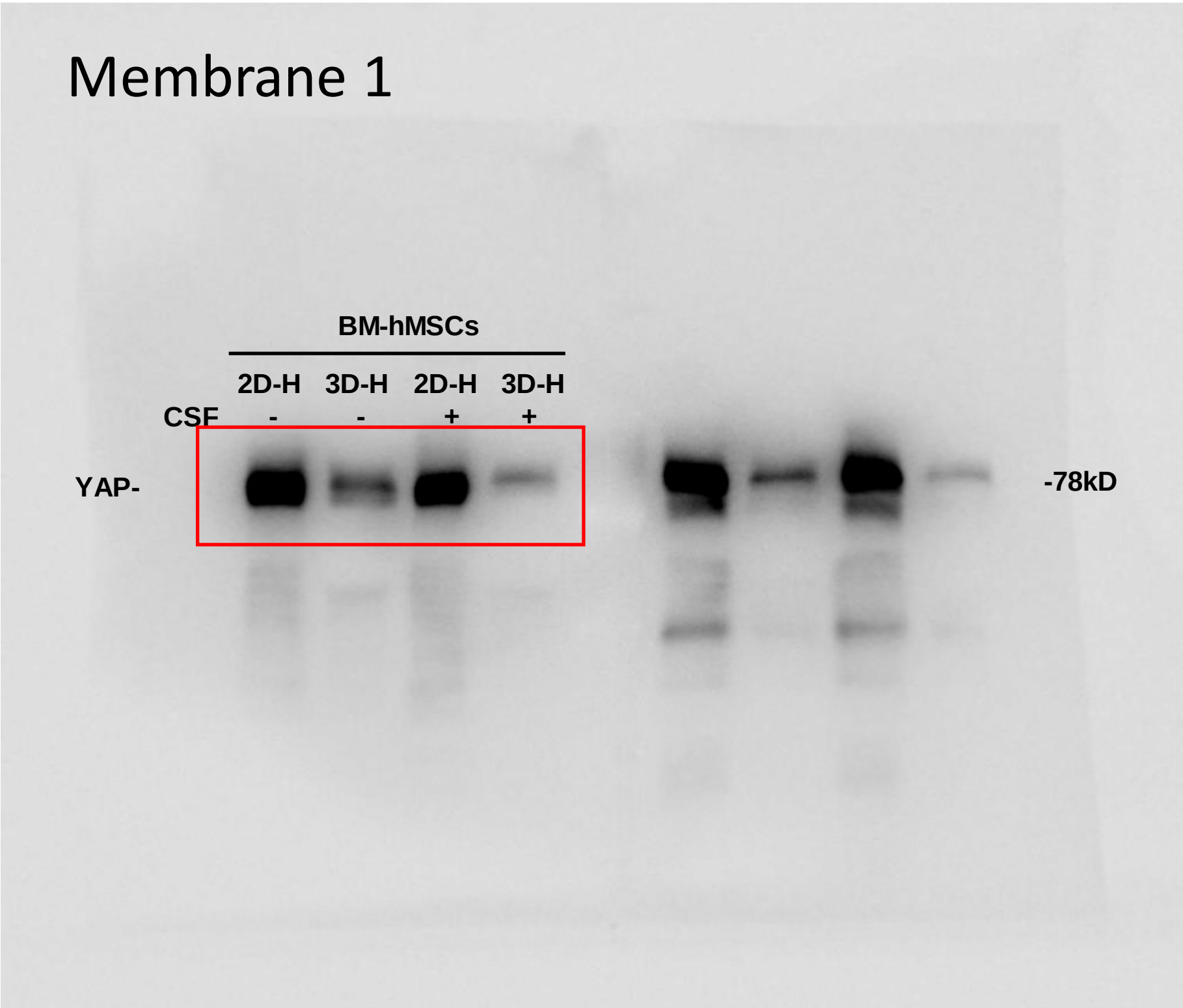

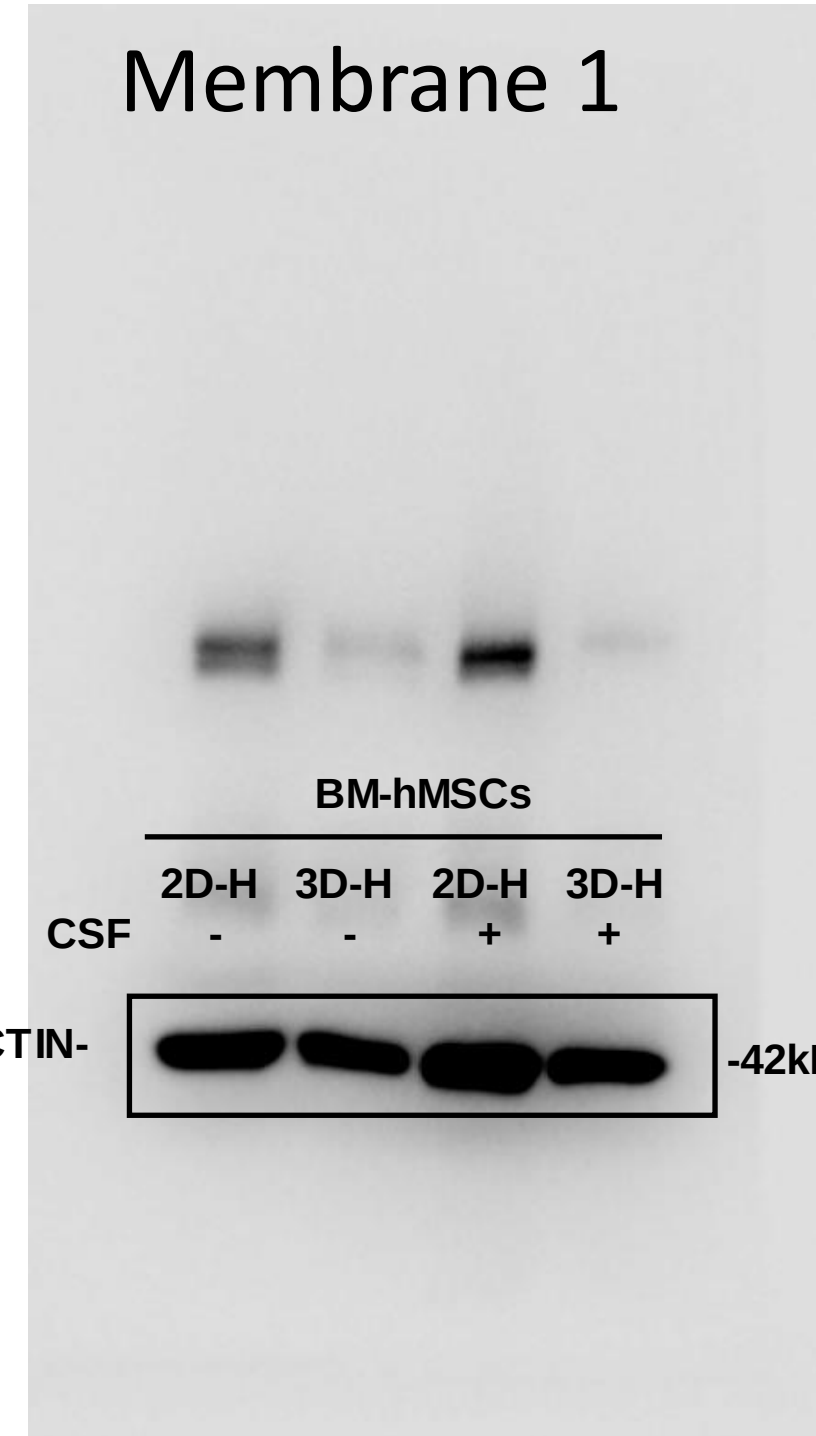

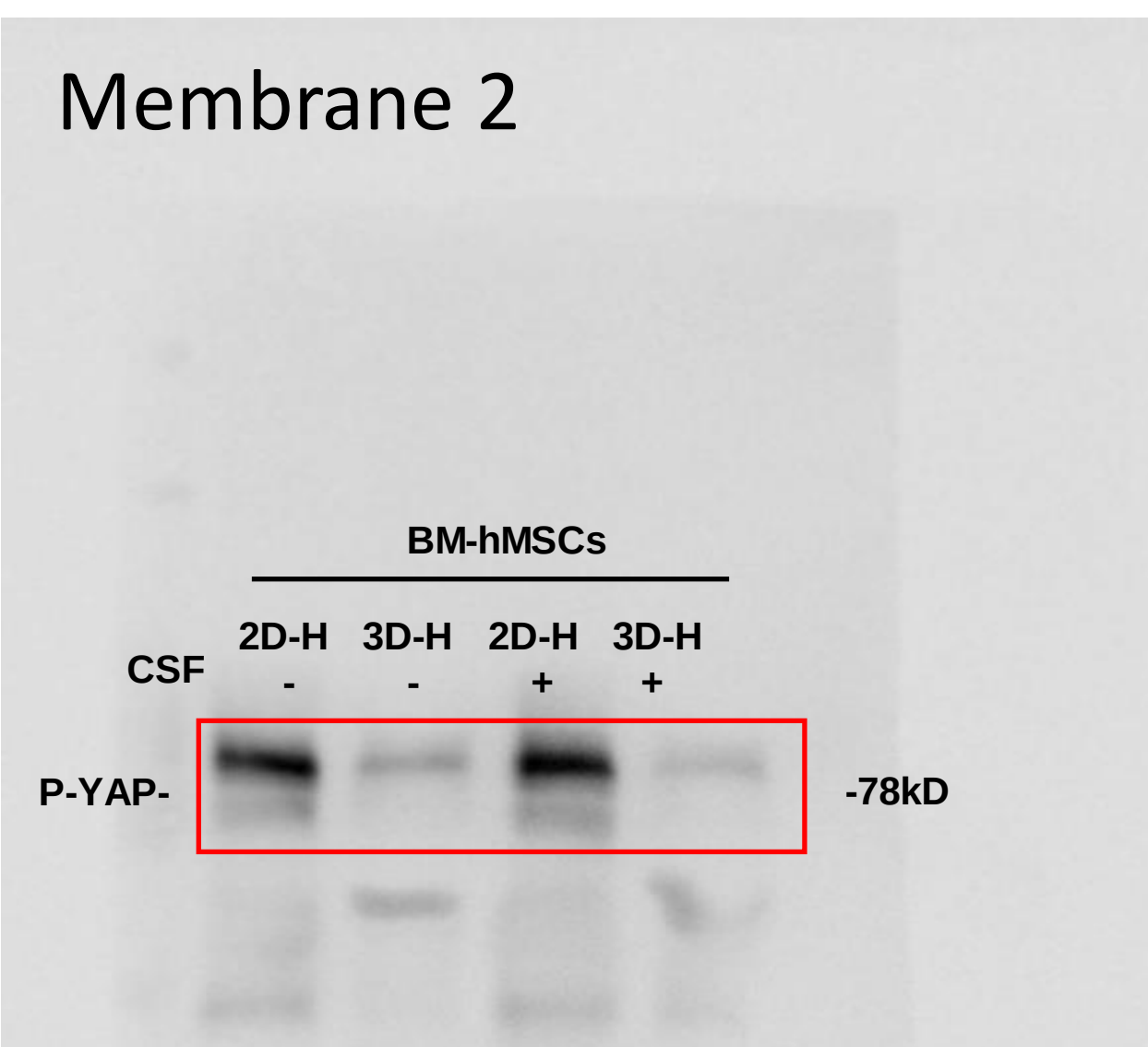

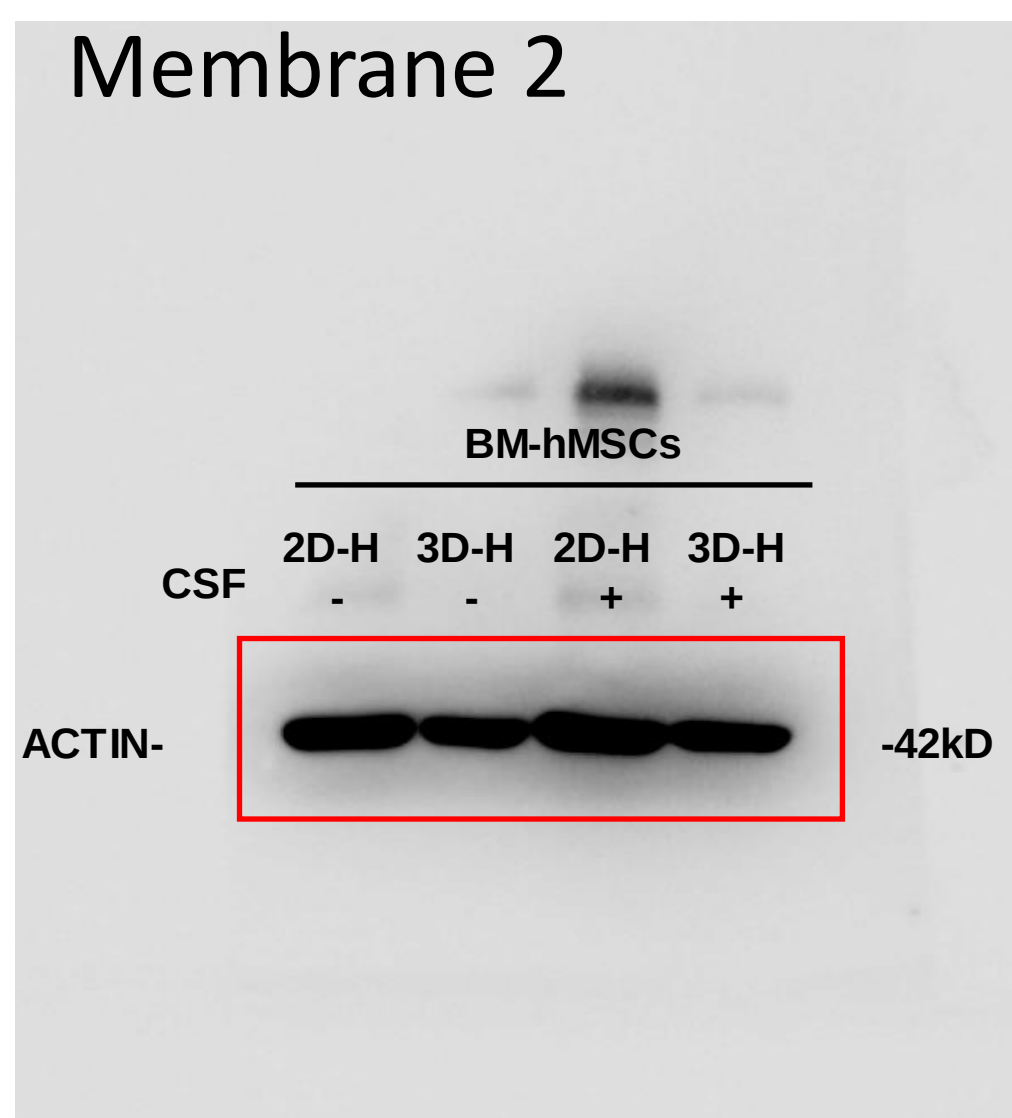

Blot presented in Figure 4A (left panel)

Original ACTIN blot from the same membrane as YAP shown in Figure 4A.

Supplementary Figure 10
Associated with Figure 4C

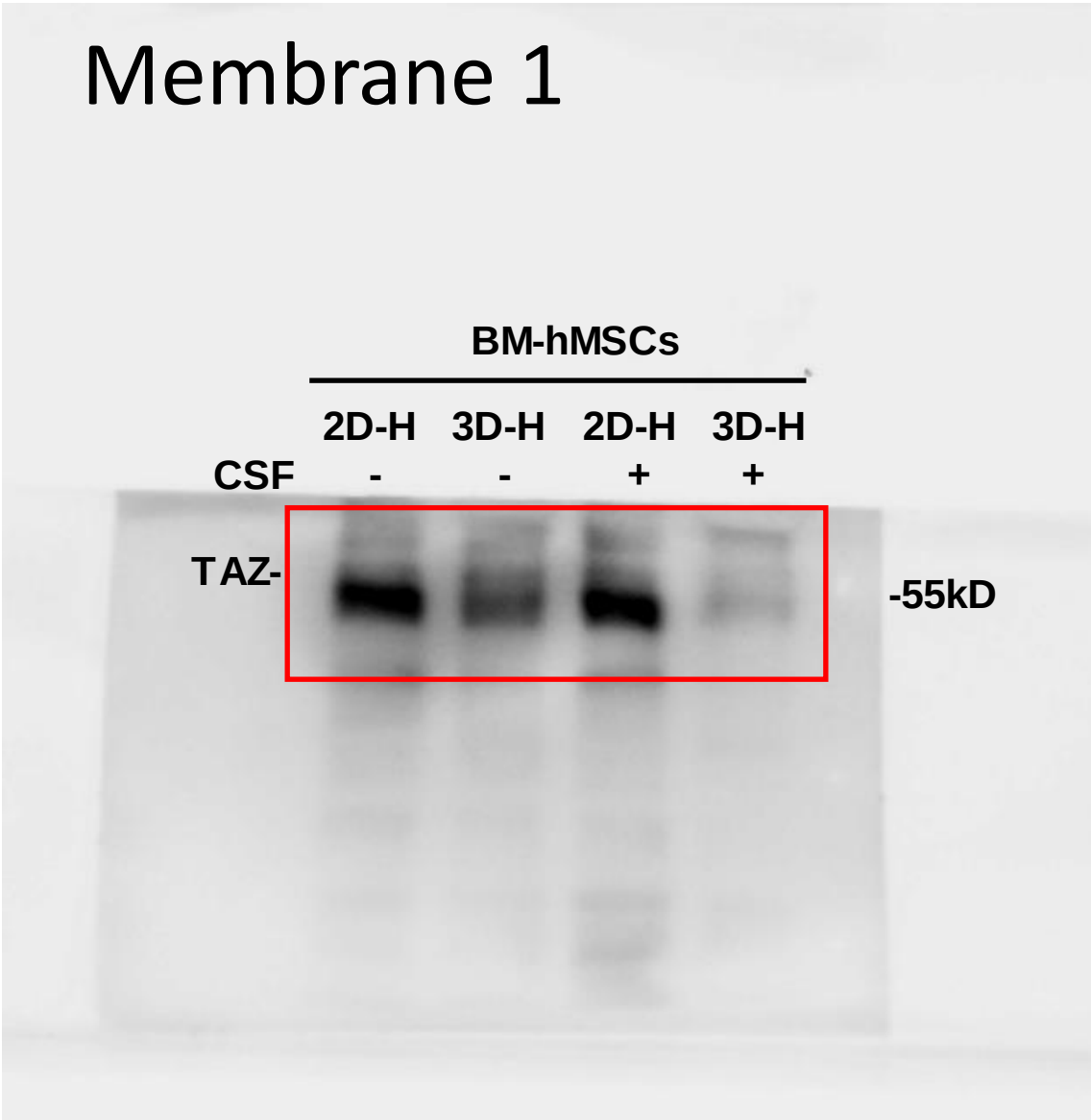

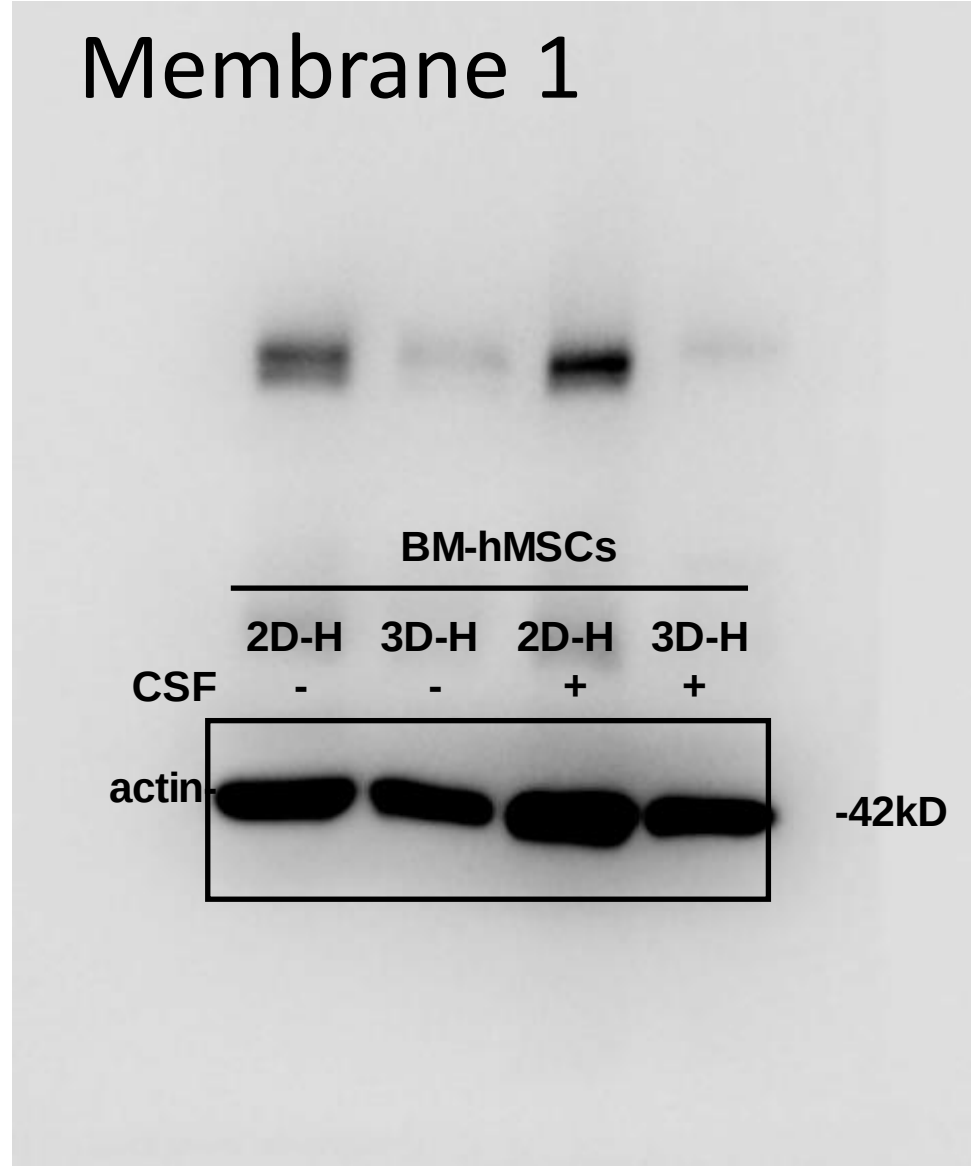

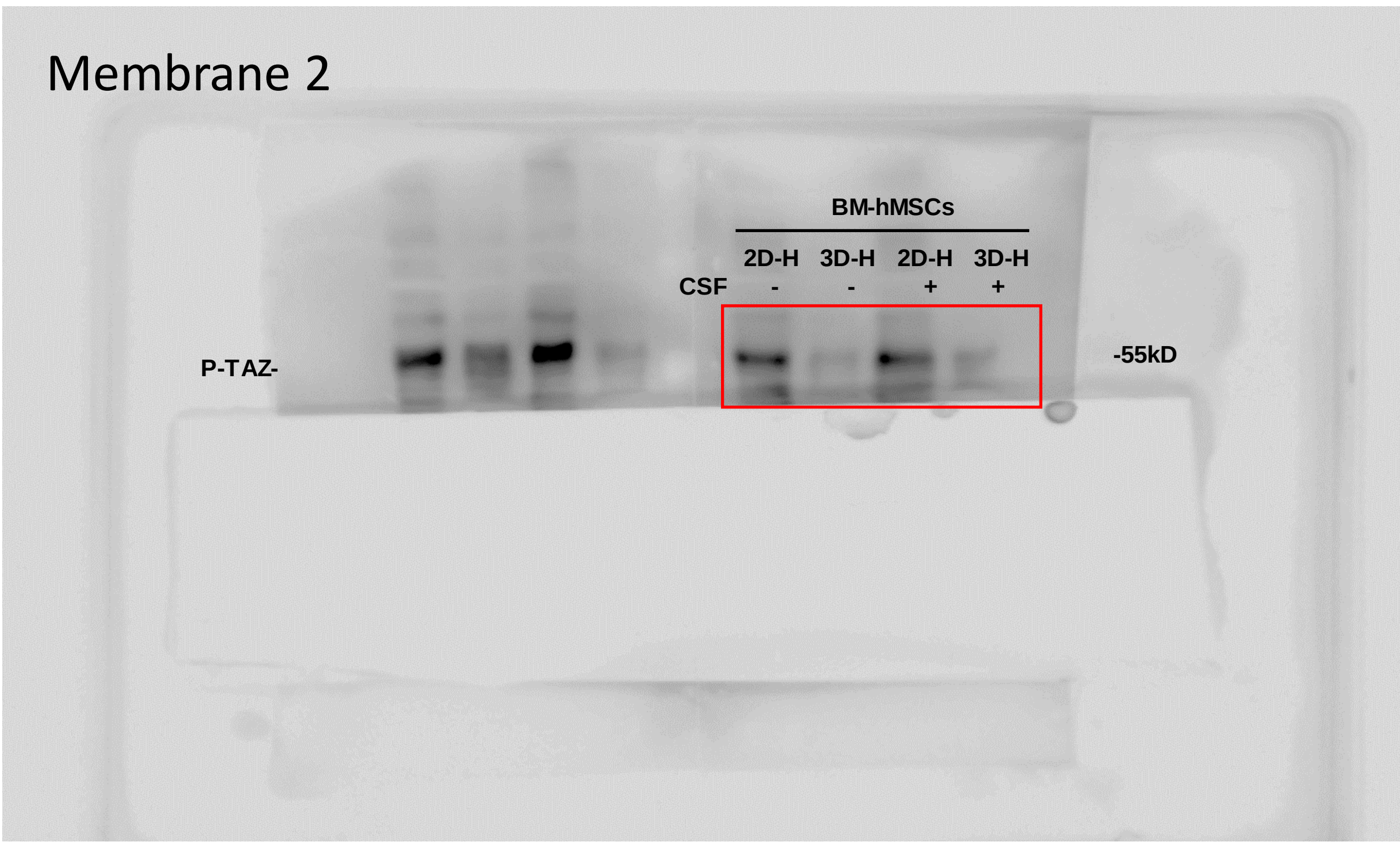

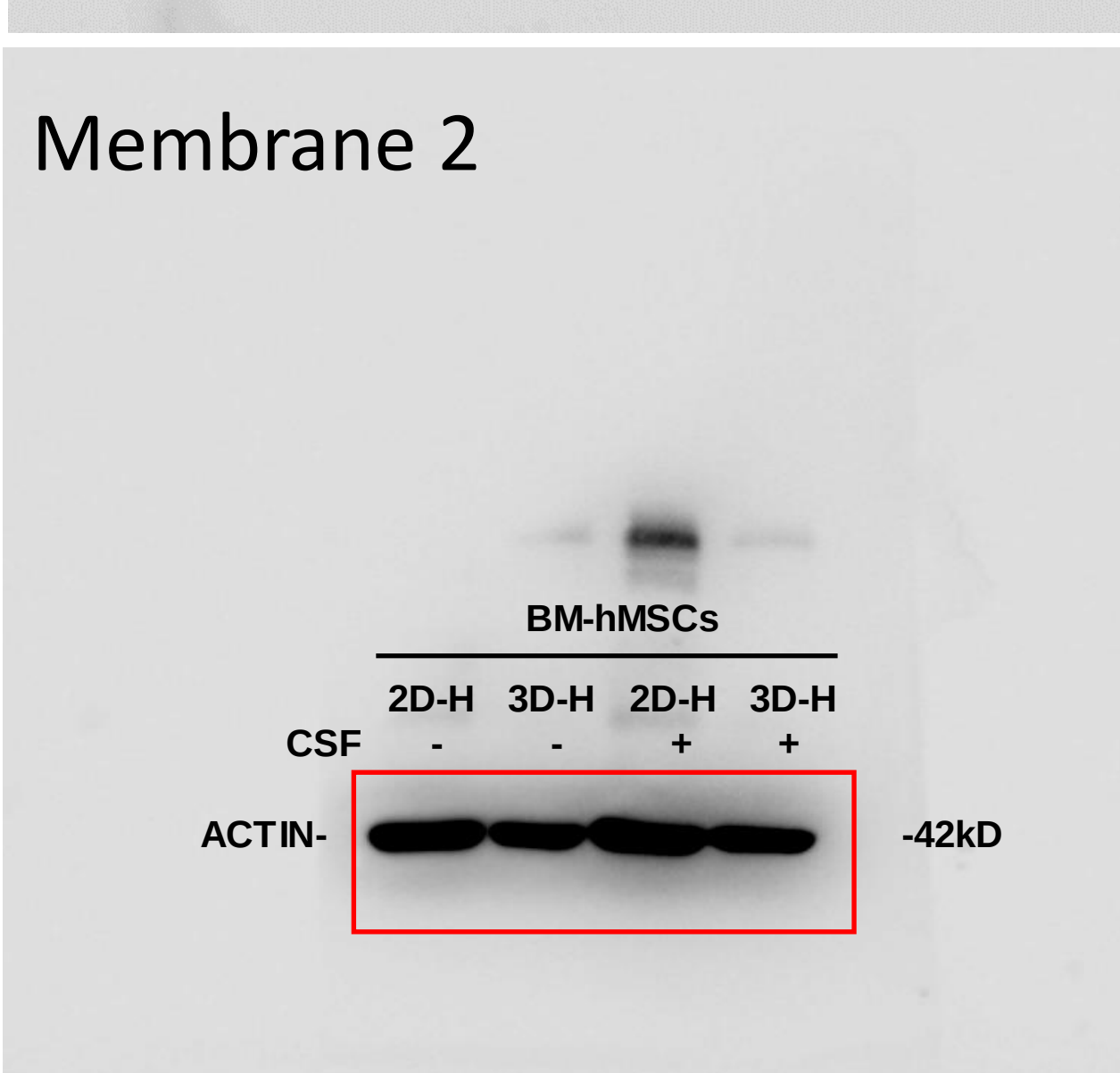

Blot presented in Figure 4C

Original ACTIN blot from the same membrane as TAZ shown in Figure 4C.

Supplementary Figure 11
Associated with Figure 5B

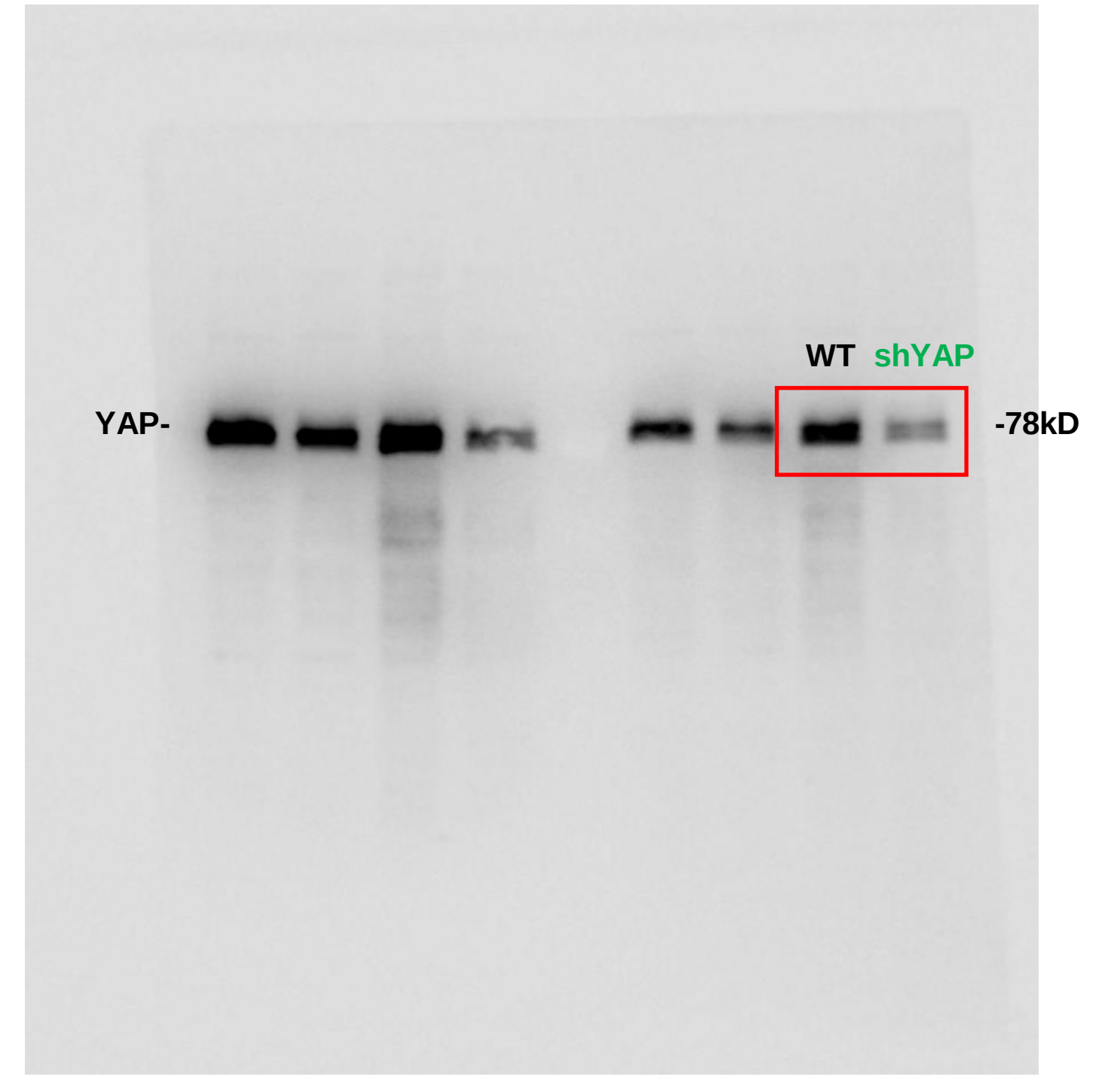

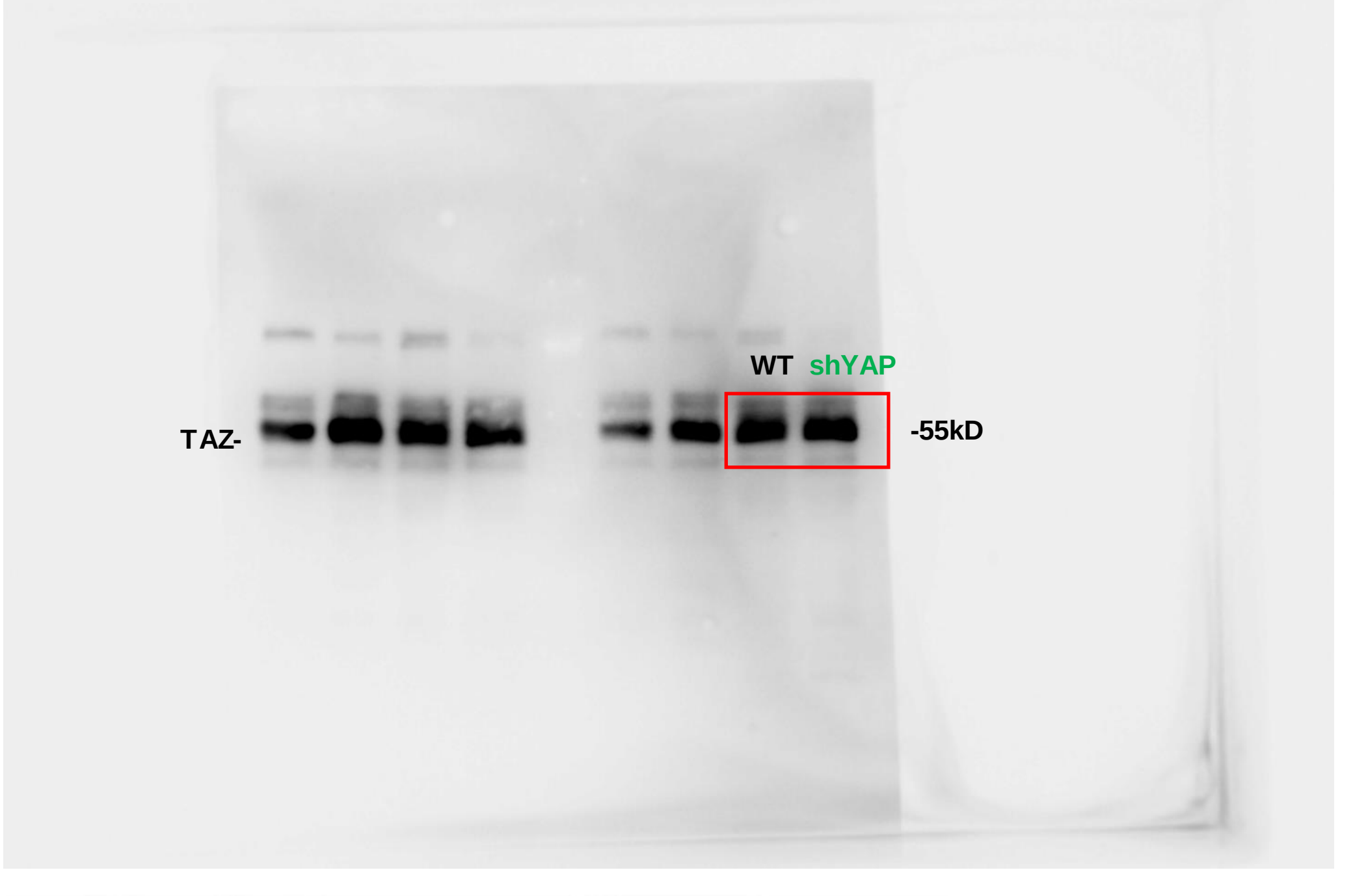

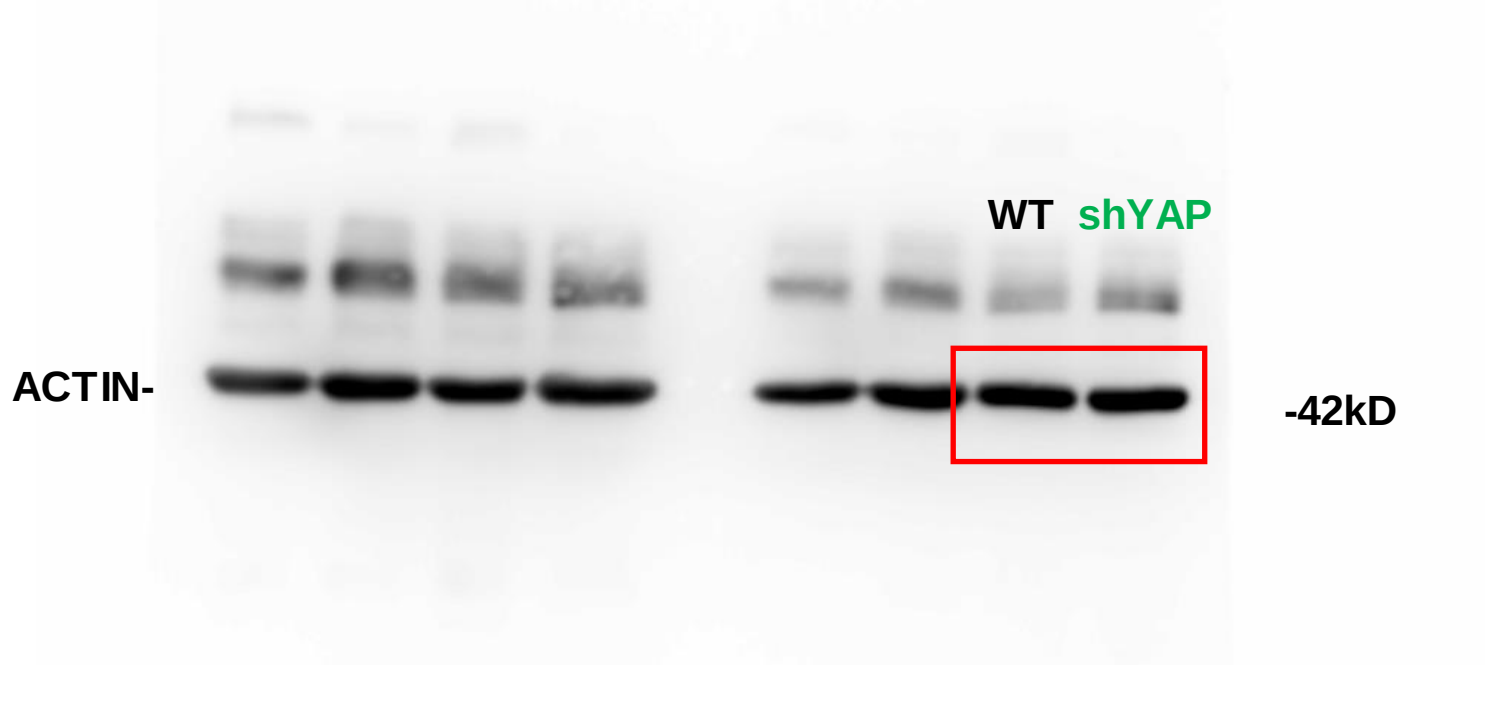

Supplementary Figure 12
Associated with Figure 5C

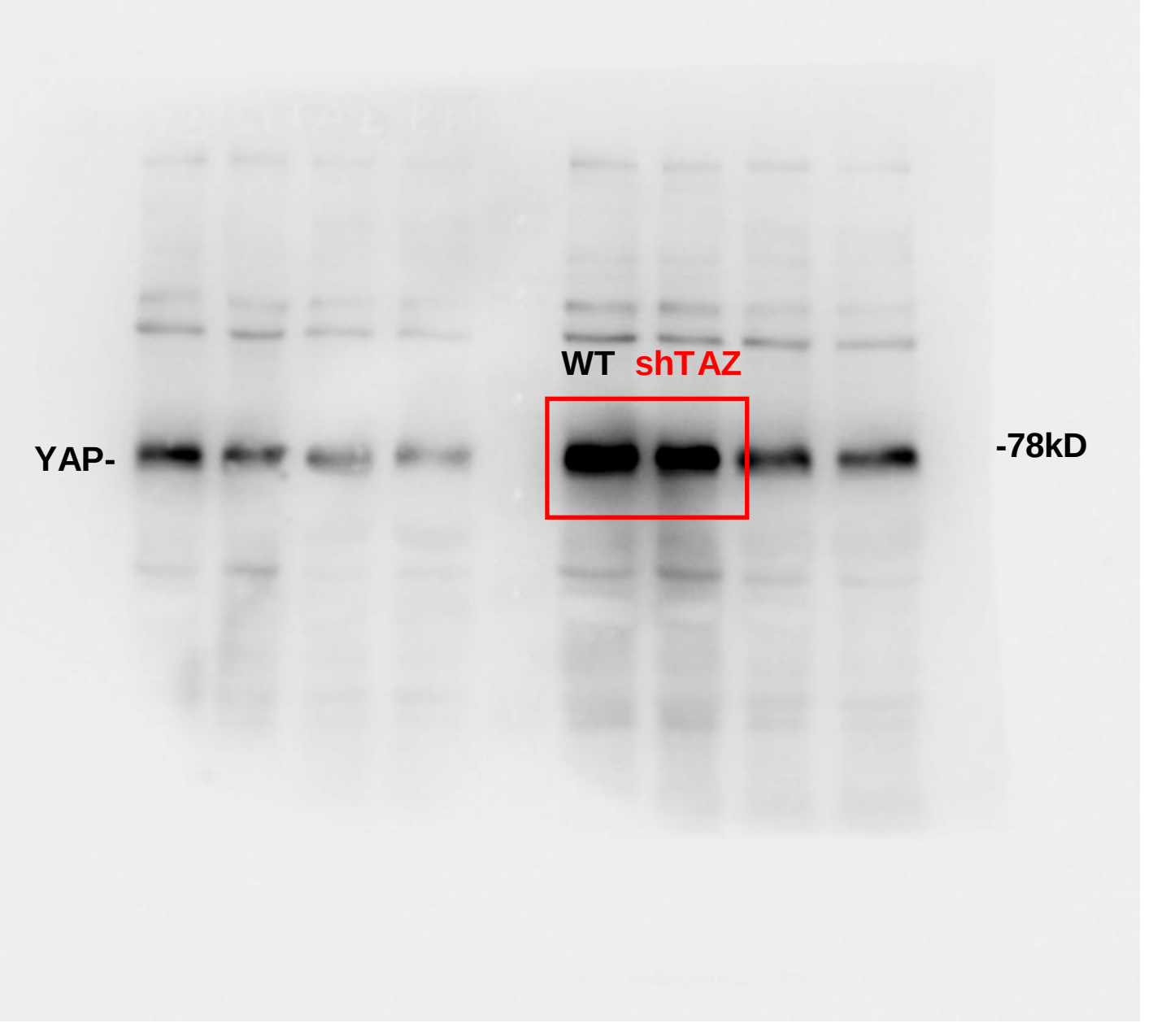

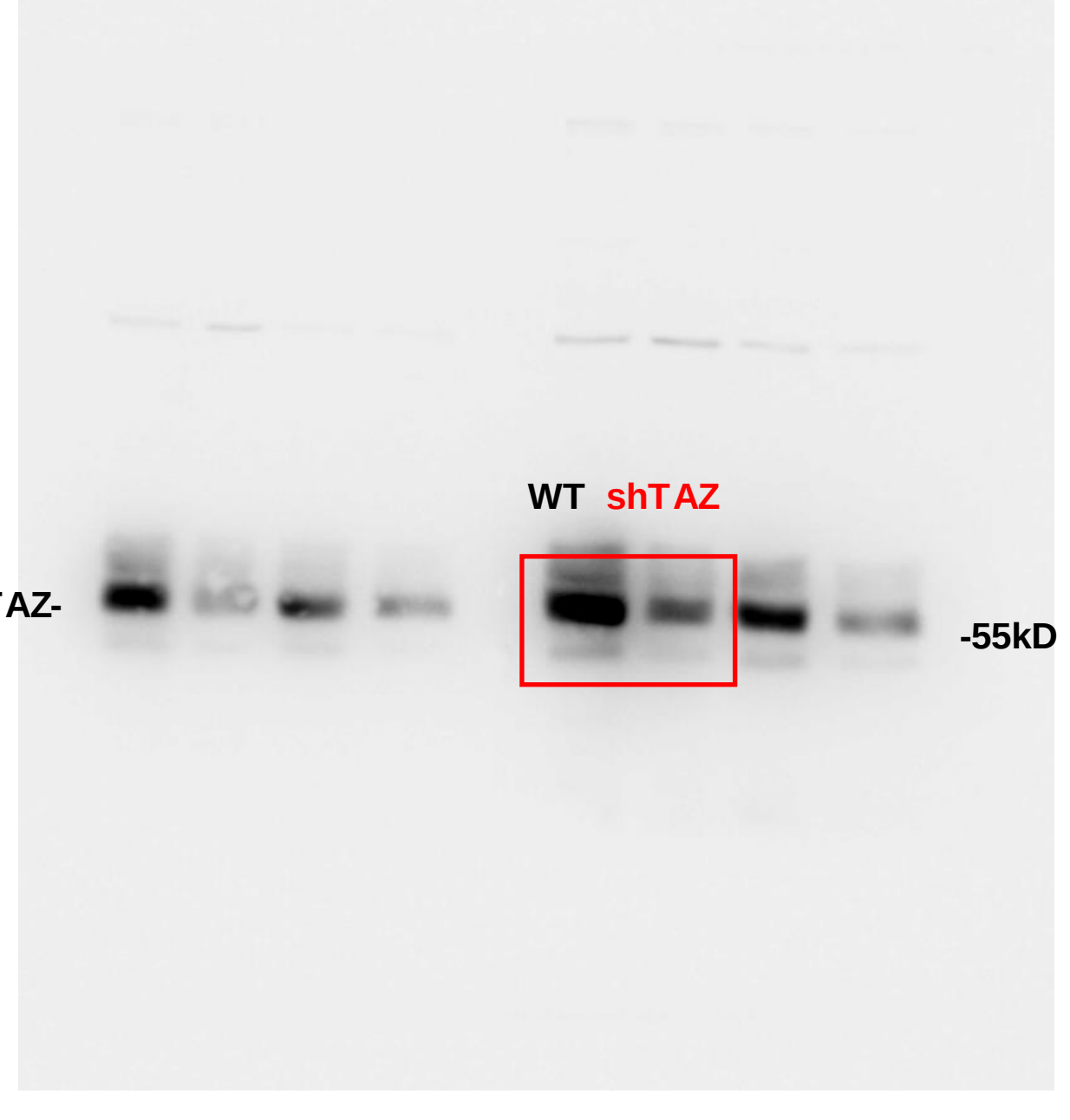

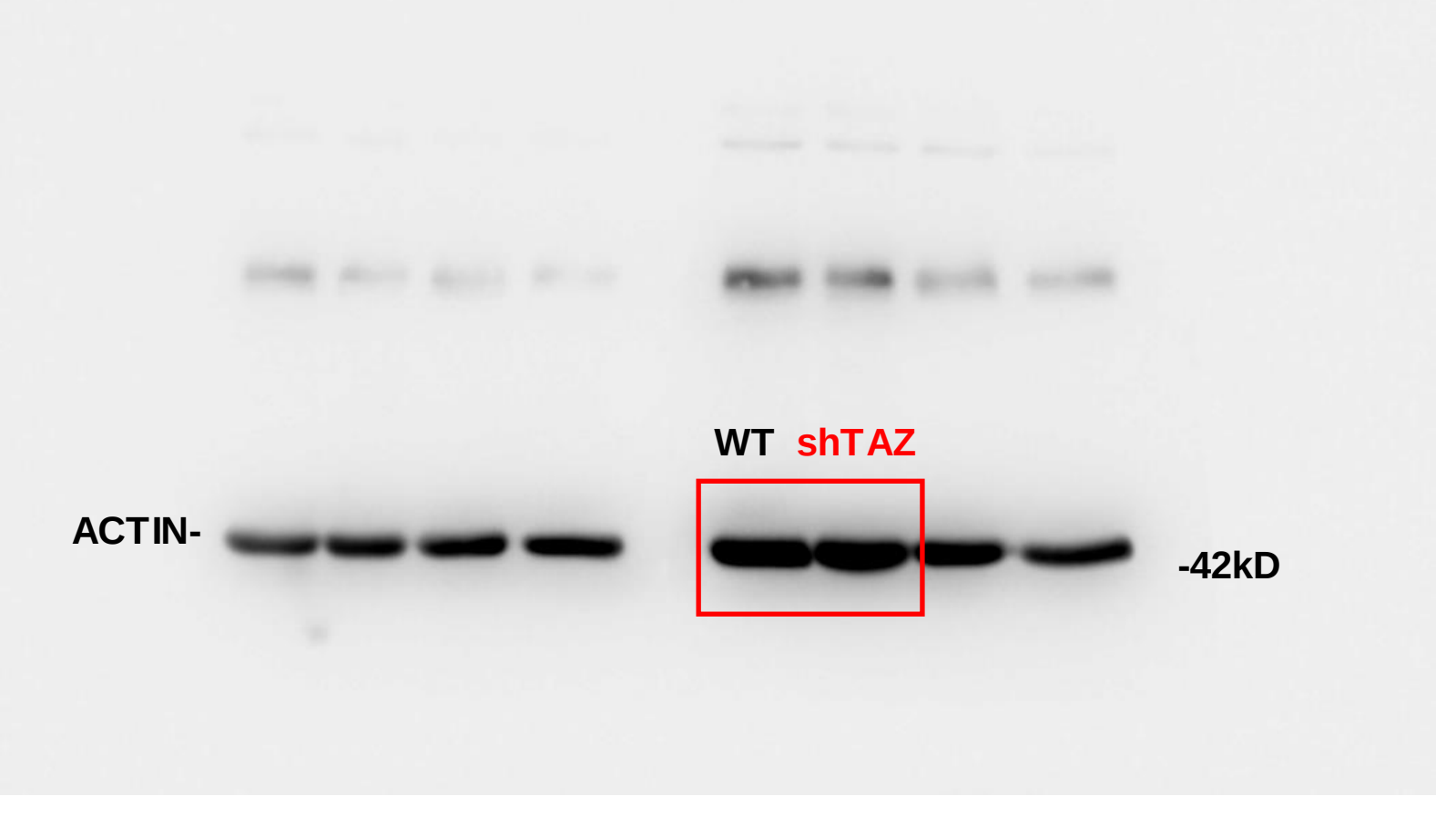

Supplementary Figure 13
Associated with supplement figure 4A left

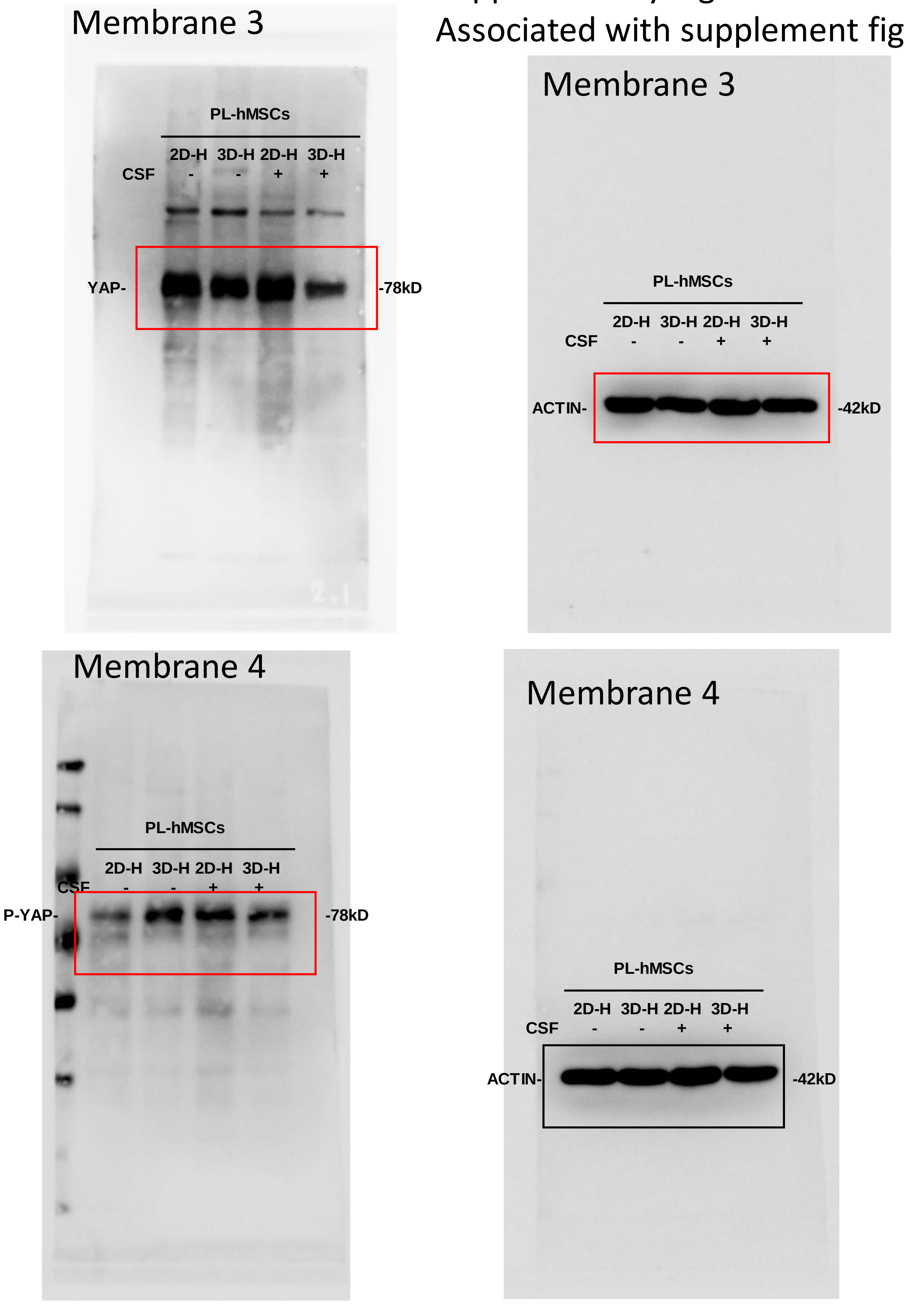

☐ (red) Blot presented in Supplementary Figure 4A (left panel)

☐ Original ACTIN blot from the same membrane of P-YAP shown in supplementary Figure 4A (left panel).

Supplementary Figure 14

Associated with supplement figure 4A right

Membrane 3

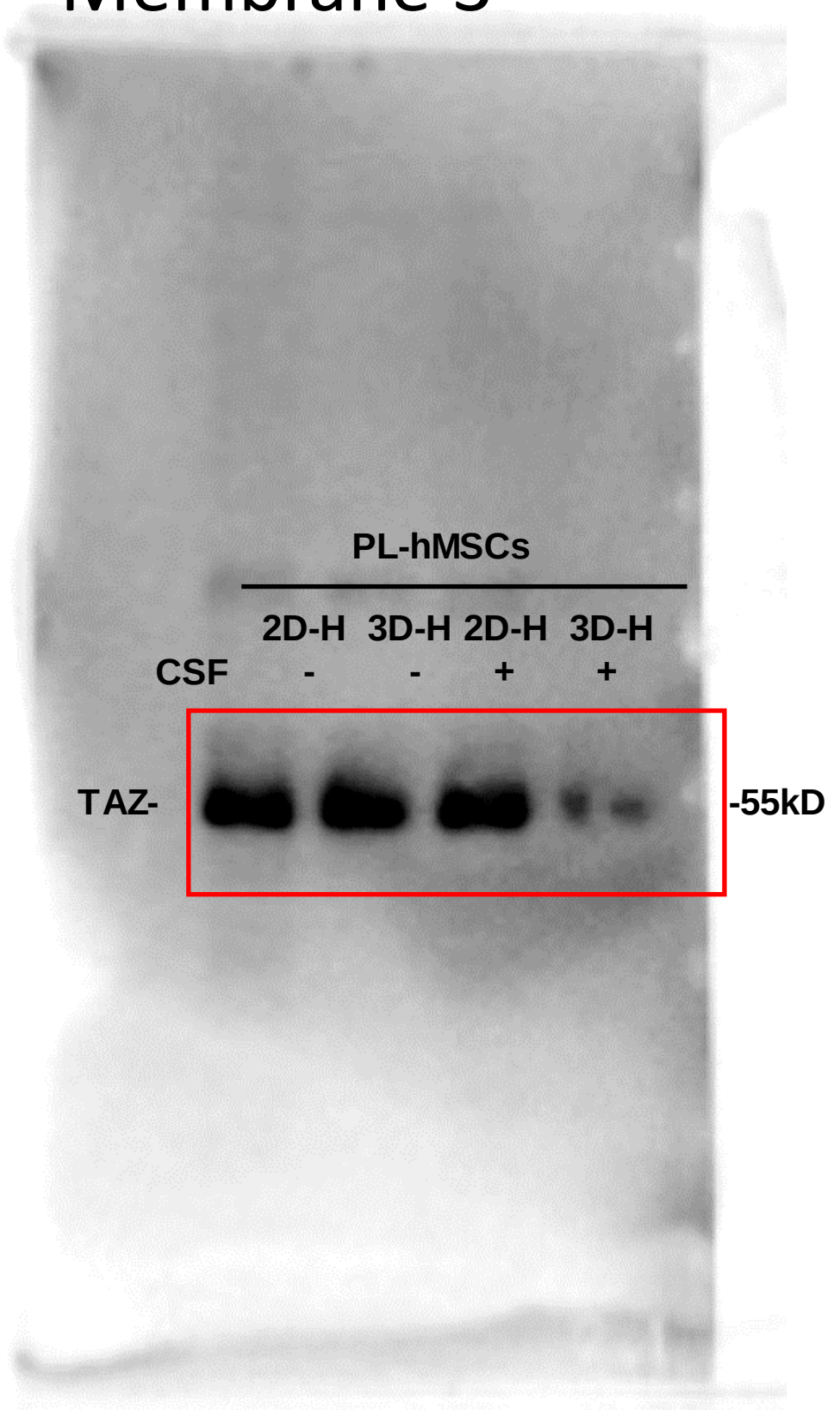

Membrane 3

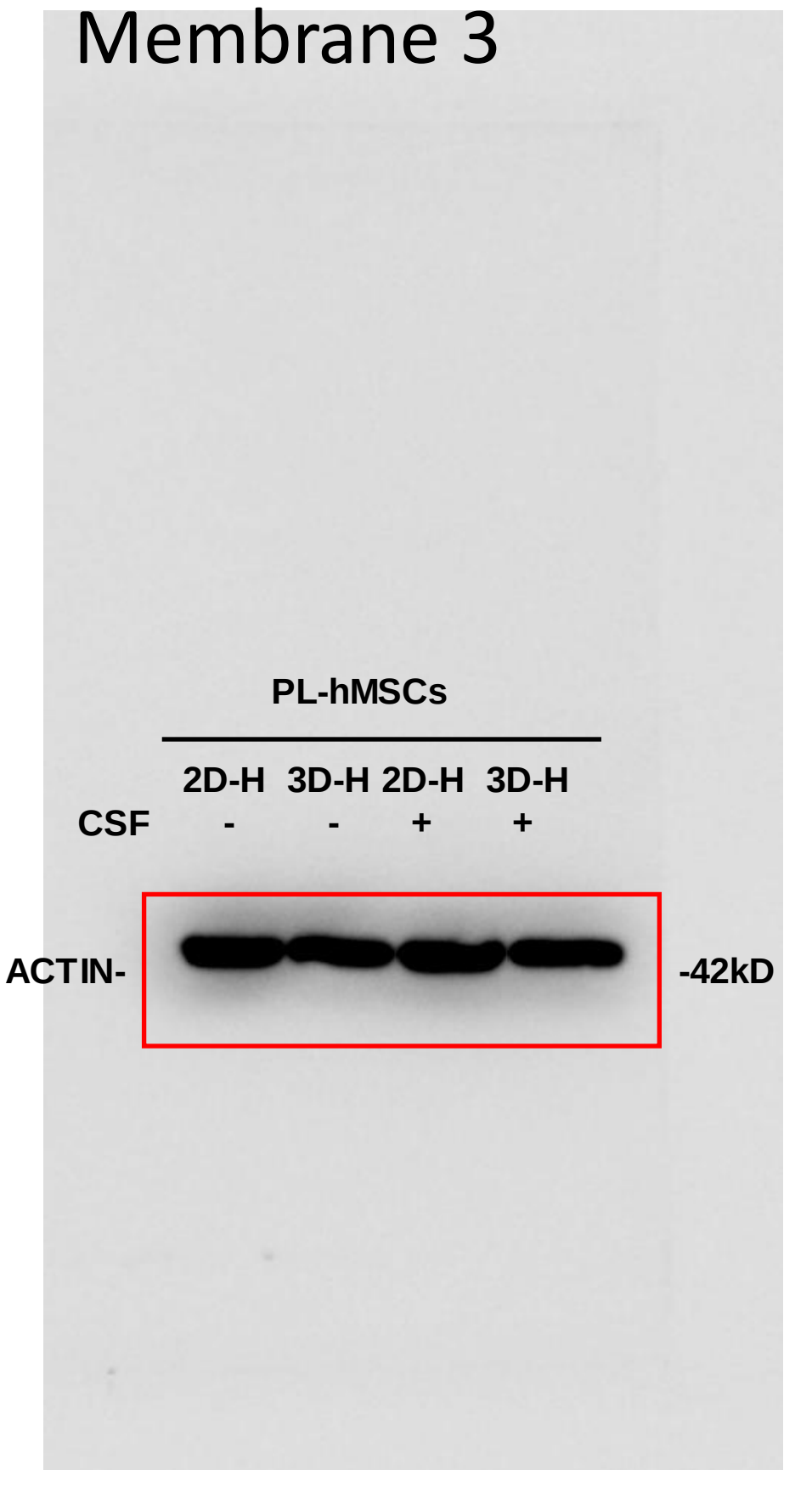

Membrane 4

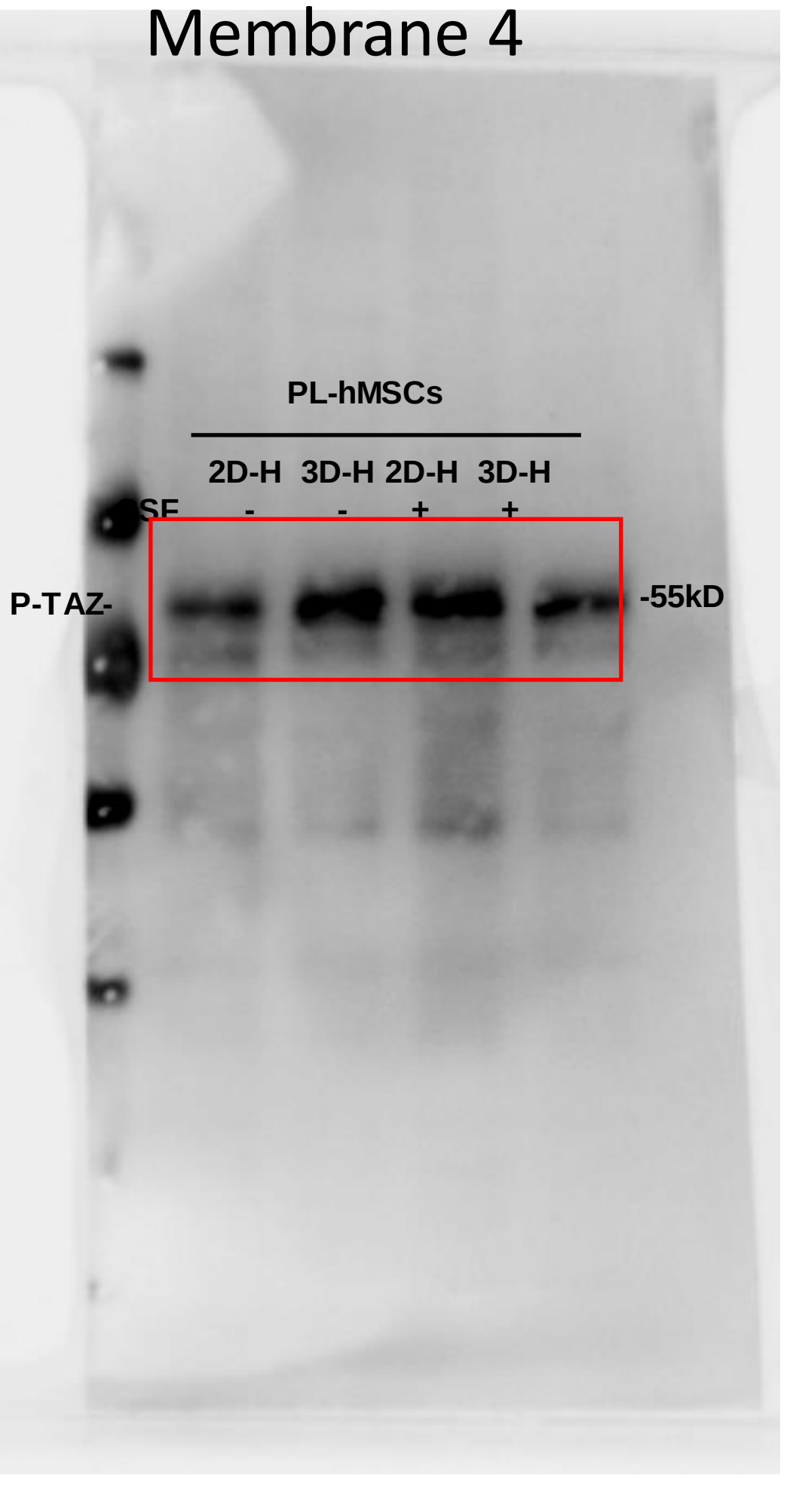

Membrane 4

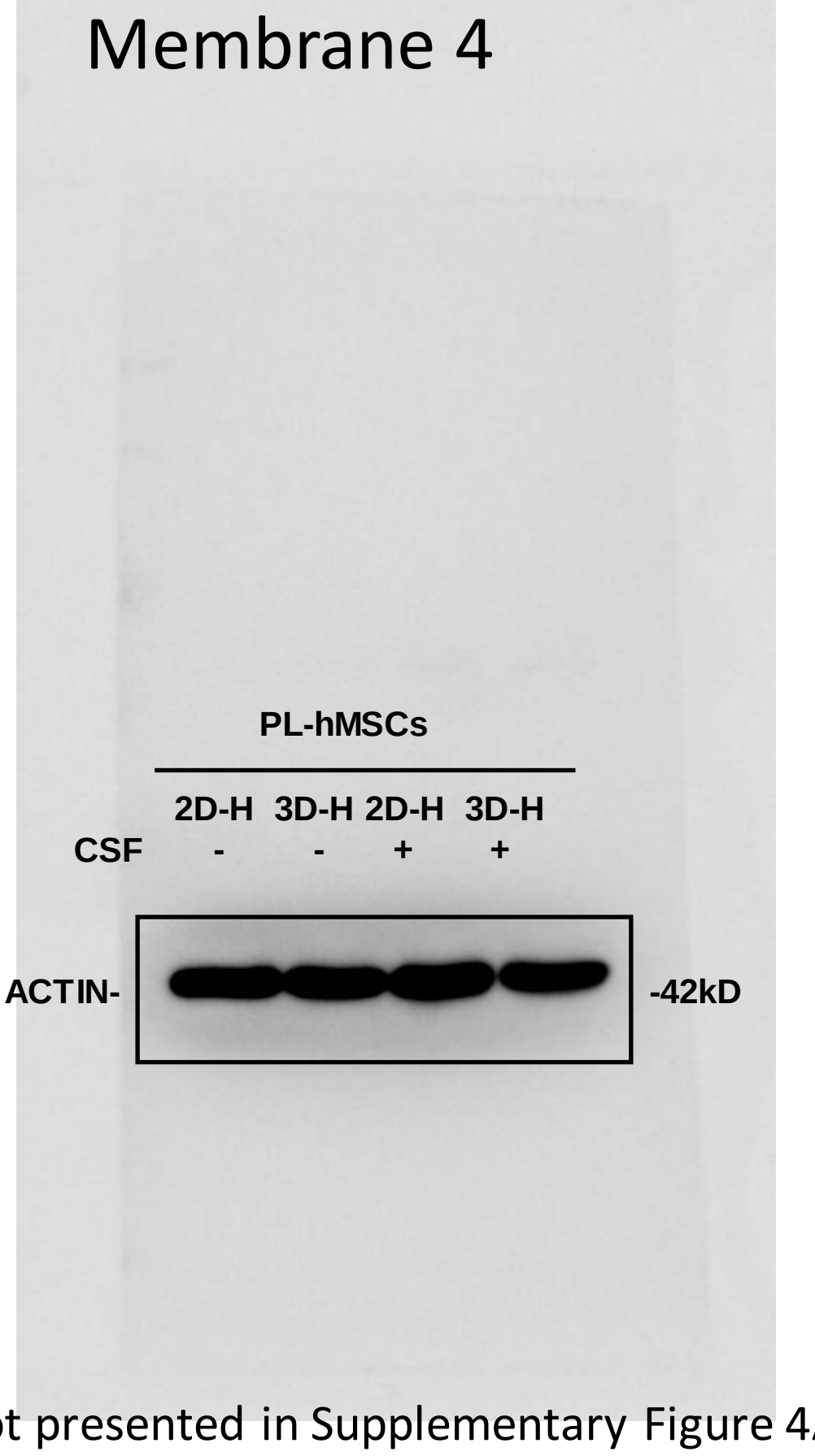

Red box: Blot presented in Supplementary Figure 4A (right panel)

Black box: Original ACTIN blot from the same membrane as P-TAZ shown in supplementary Figure 4A (right panel).

Supplementary Figure 15
Associated with supplement figure 4B left

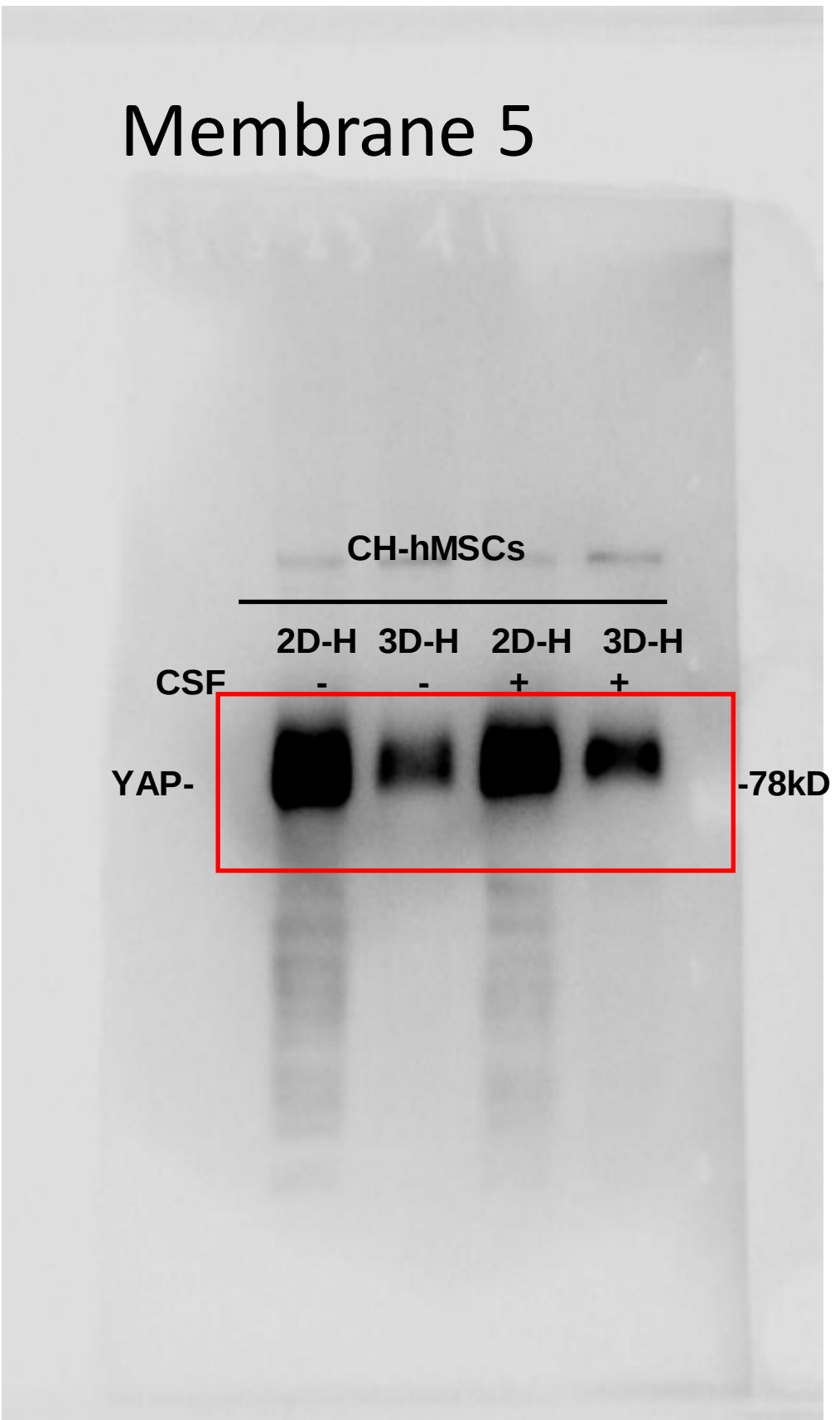

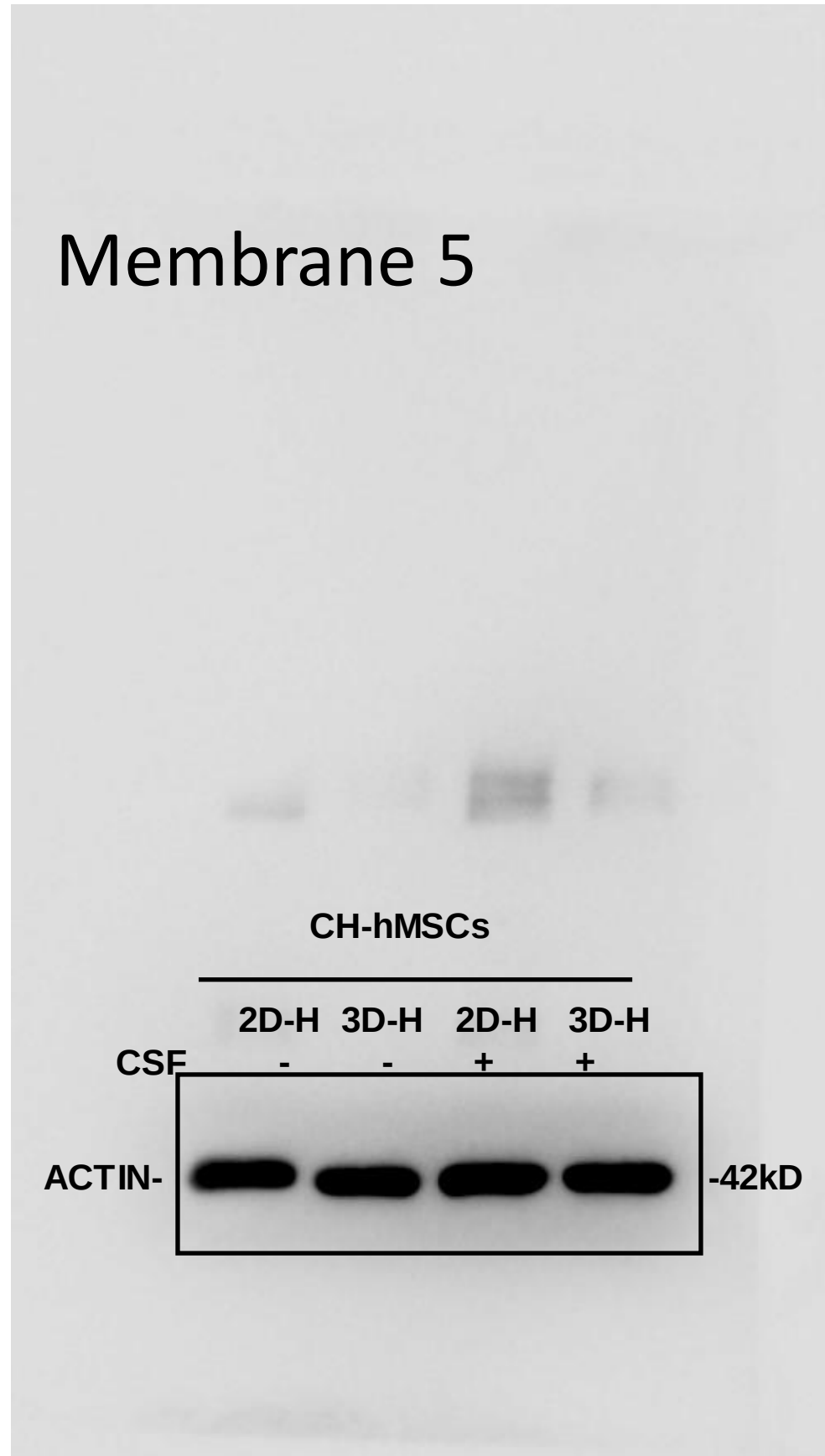

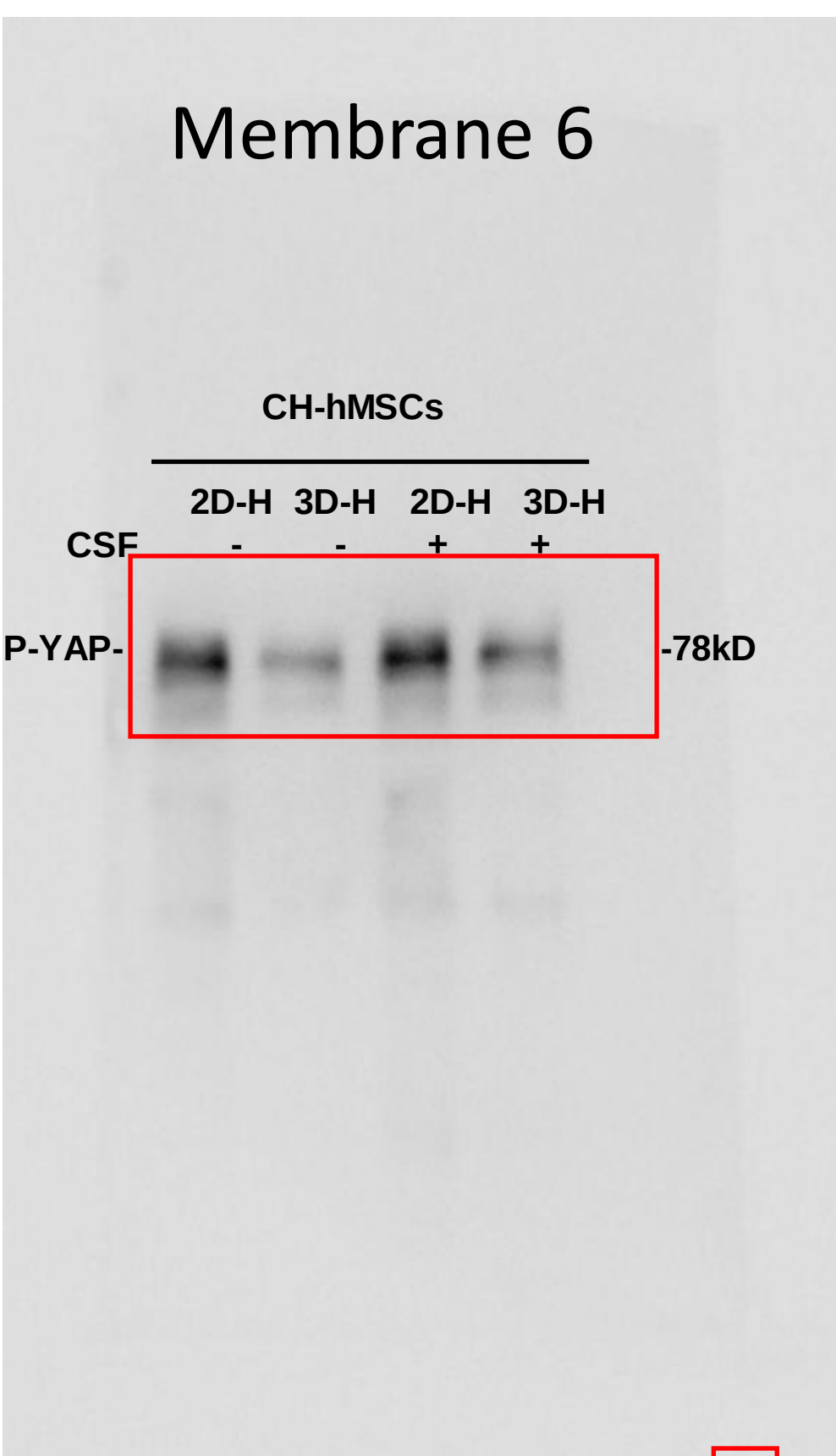

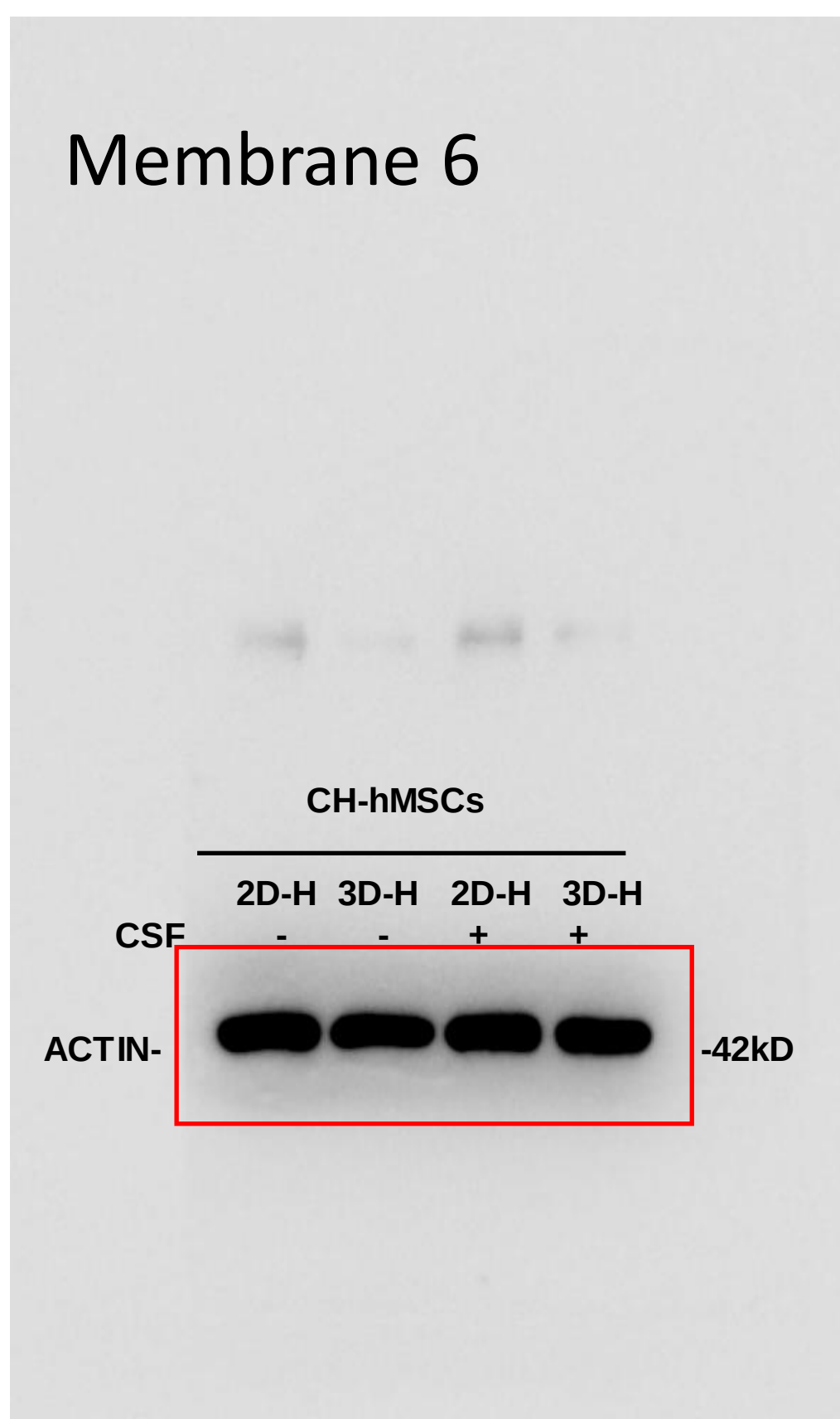

Blot presented in Supplementary Figure 4B (left panel)

Original ACTIN blot from the same membrane of YAP shown in Supplementary Figure 4B (left panel).

Supplementary Figure 16
Associated with to supplement figure 4B right

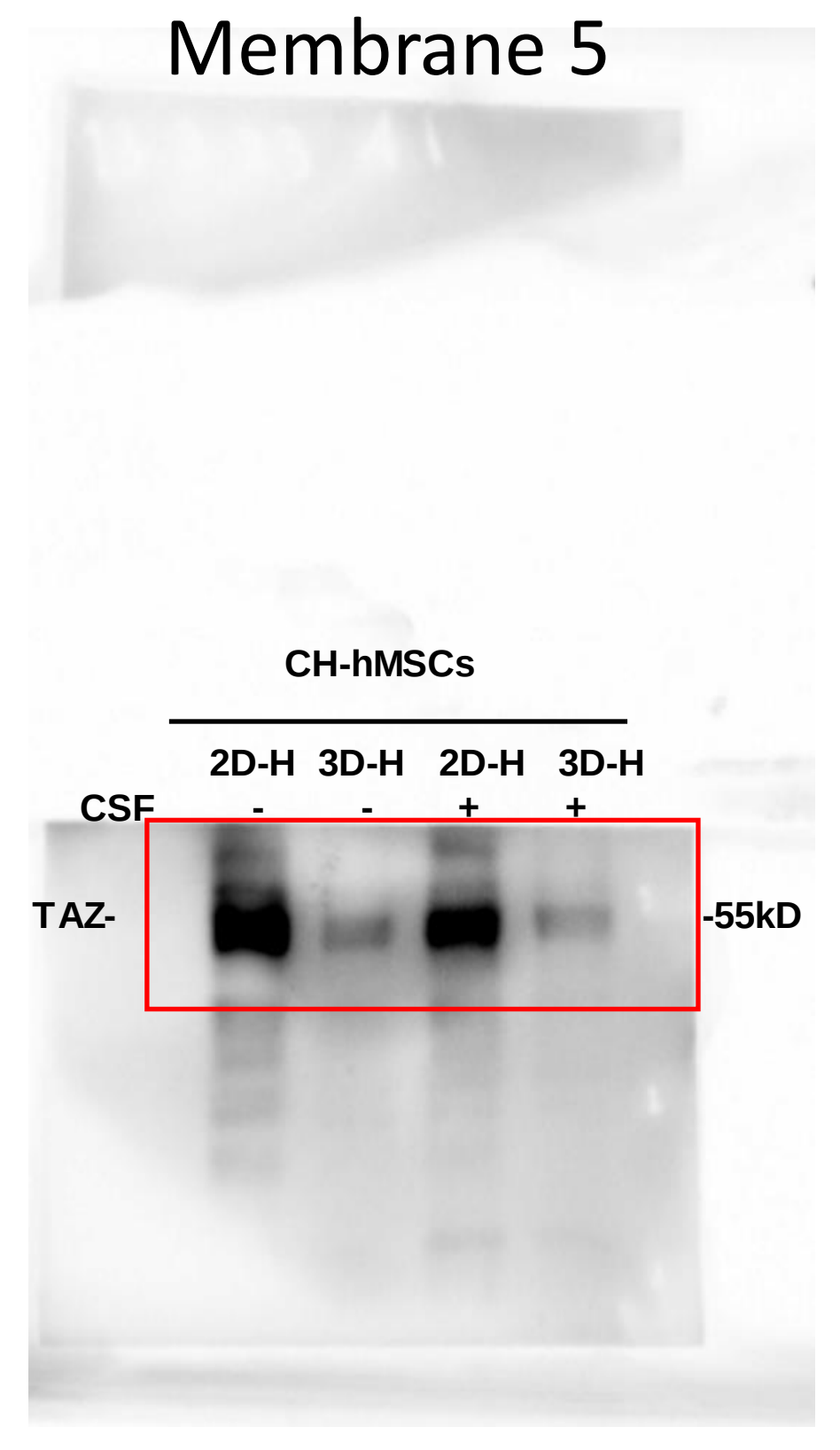

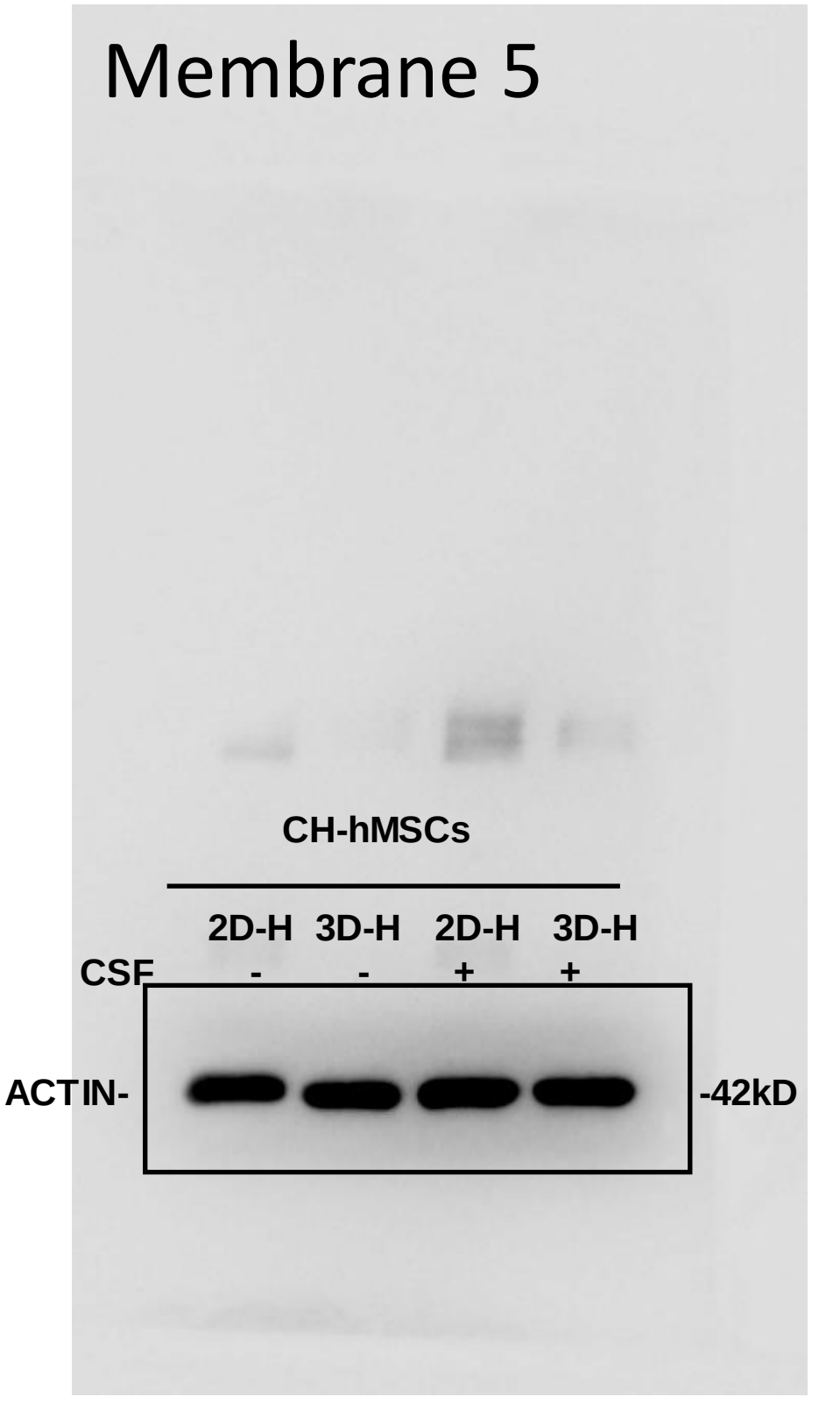

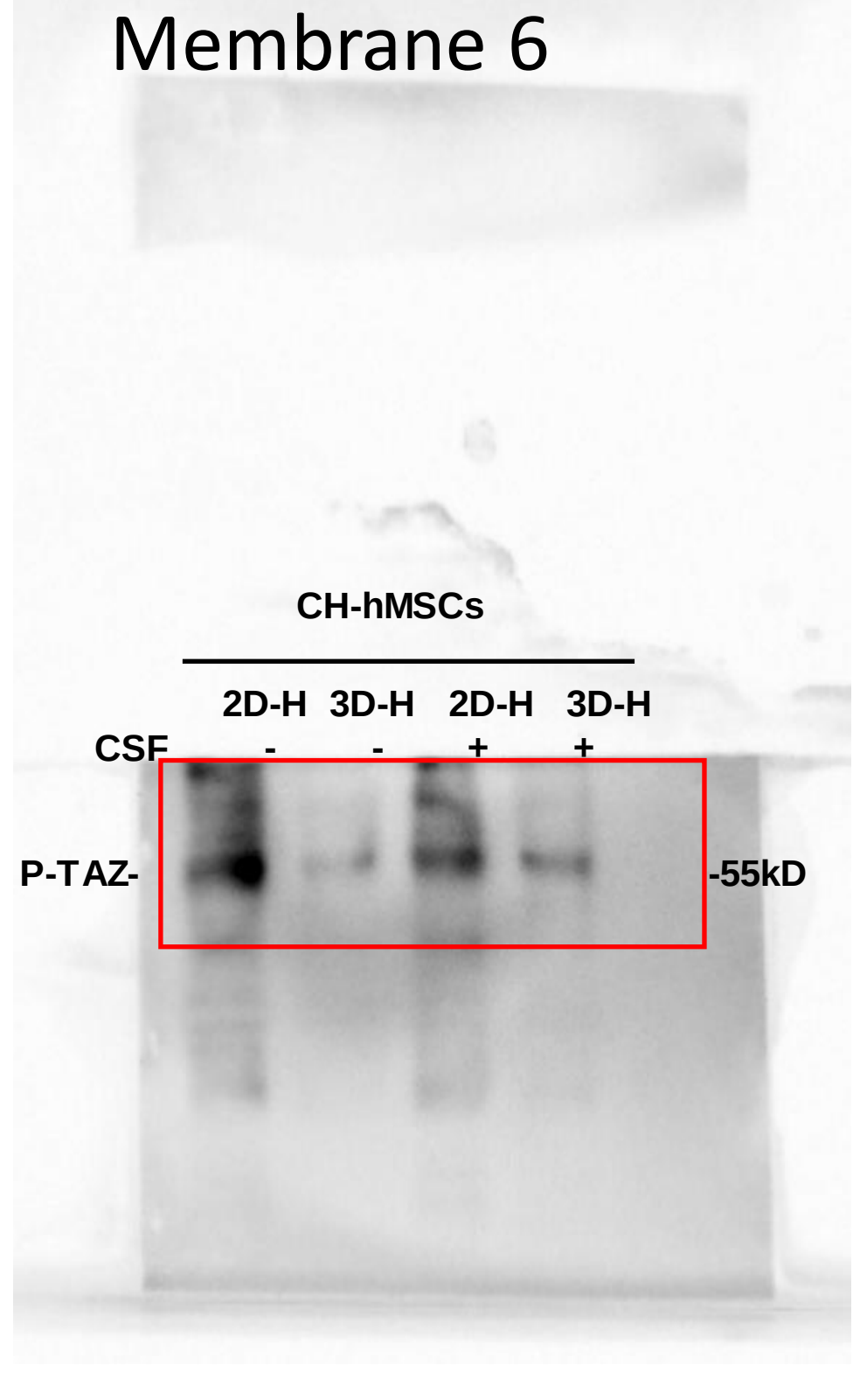

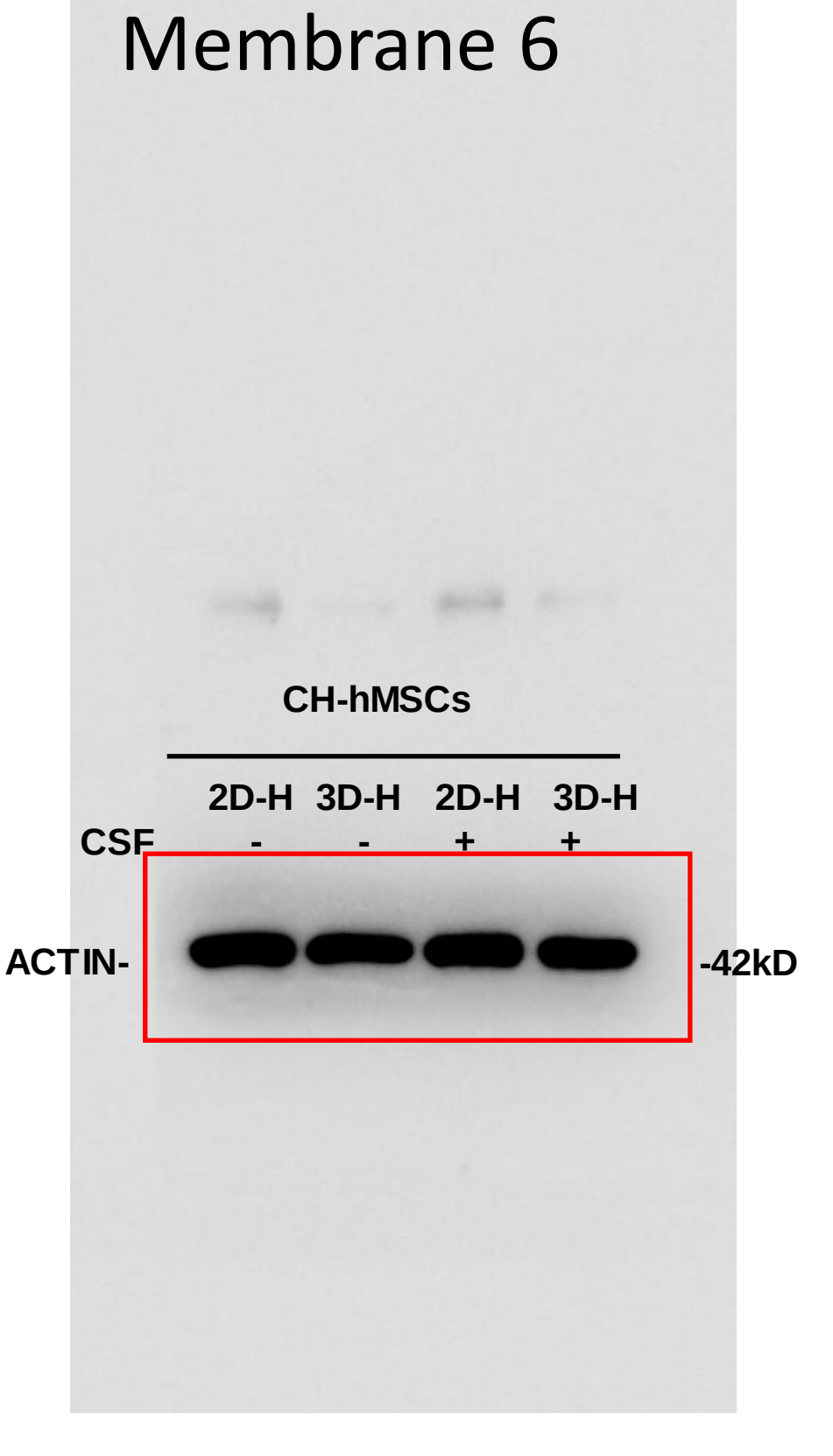

Blot presented in Supplementary Figure 4B (right panel)

Original ACTIN blot from the same membrane of YAZ shown in Supplementary Figure 4B (right panel).
