# Supplementary material for: Hippo Pathway Regulation and Microenvironmental Cues Governing Human MSC Transition to Cancer‐Associated Fibroblasts
Source: J Cell Physiol. 2026 Aug 5;241(8):e70219. doi: 10.1002/jcp.70219 (PMC13440140; doi:10.1002/jcp.70219)
Supplement: Supplementary file 2 [file JCP-241-0-s001.docx]

**Supplementary Table 1.** Primers used for quantitative real-time polymerase chain reaction

| **Gene** | **Forward primer sequences** | **Reverse primer sequences** |
| --- | --- | --- |
| *GAPDH* | 5′- TCATGGGTGTGAACCATGAGAA-3′ | 5′-GGCATGGACTGTGGTCATGAG-3′ |
| *IL1A* | 5′-GTAAGCTATGGCCCACTCCA-3′ | 5′-AGGTGCTGACCTAGGCTTGA-3′ |
| *CSF3* | 5′-CACTCTGGACAGTGCAGGAA-3′ | 5′-AGAGAGTGTCCGAGCAGCA-3′ |
| *IL6-F* | 5′-CACAGACAGCCACTCACCTC-3′ | 5′-AGTGCCTCTTTGCTGCTTTC-3′ |
| *MYL9* | 5′-GGATGTGATTCGCAACGCCTTTG-3′ | 5′-CGGTACATCTCGTCCACTTCCT-3′ |
| *TAGLN* | 5′-TCCAGGTCTGGCTGAAGAATGG-3′ | 5′-CTGCTCCATCTGCTTGAAGACC-3′ |
| *CCN2* | 5′- CCTGGTCCAGACCACAGAGT-3′ | 5′-TGGAGATTTTGGGAGTACGG-3′ |
