# Supplementary material for: Hippo Pathway Regulation and Microenvironmental Cues Governing Human MSC Transition to Cancer‐Associated Fibroblasts
Source: J Cell Physiol. 2026 Aug 5;241(8):e70219. doi: 10.1002/jcp.70219 (PMC13440140; doi:10.1002/jcp.70219)
Supplement: Supplementary file 3 [file JCP-241-0-s003.docx]

**Supplementary Table 2:** Mycoplasma test result of uE6E7T-3

| **Cell line** | **Reading A** | **Reading B** | **Ratio (B/A)** | **Interpretation** |
| --- | --- | --- | --- | --- |
| Positive control | 72499 | 3179224 | 43.85 | Contamination |
| Negative control | 109448 | 34649 | 0.31657 | Negative |
| uE6E7T-3 | 13399 | 11019 | 0.82237 | Negative |

**Ratio Interpretation**

<1	Negative for mycoplasma

1-1.2	Quarantine cells and retest in 24 or 48 h

>1.2	Mycoplasma contamination

**Note:** Divide read B by read A to produce the ratio
